# Unconstrained Precision Mitochondrial Genome Editing with αDdCBEs

**DOI:** 10.1089/hum.2024.073

**Published:** 2024-10-14

**Authors:** Santiago R. Castillo, Brandon W. Simone, Karl J. Clark, Patricia Devaux, Stephen C. Ekker

**Affiliations:** ^1^ Virology and Gene Therapy Graduate Program, Mayo Clinic, Rochester, Minnesota, USA; ^2^ Department of Molecular Medicine, Mayo Clinic, Rochester, Minnesota, USA; ^3^ Department of Biochemistry and Molecular Biology, Mayo Clinic, Rochester, Minnesota, USA; ^4^ Department of Pediatrics and Department of Molecular Biosciences, Dell Medical School, The University of Texas at Austin, Austin, Texas, USA.

**Keywords:** mitochondrial base editing, precision genome engineering, mitochondrial DNA (mtDNA), transcription activator-like effectors (TALEs), DddA-derived cytosine base editors (DdCBEs)

## Abstract

DddA-derived cytosine base editors (DdCBEs) enable the targeted introduction of C•G-to-T•A conversions in mitochondrial DNA (mtDNA). DdCBEs work in pairs, with each arm composed of a transcription activator-like effector (TALE), a split double-stranded DNA deaminase half, and a uracil glycosylase inhibitor. This pioneering technology has helped improve our understanding of cellular processes involving mtDNA and has paved the way for the development of models and therapies for genetic disorders caused by pathogenic mtDNA variants. Nonetheless, given the intrinsic properties of TALE proteins, several target sites in human mtDNA are predicted to remain out of reach to DdCBEs and other TALE-based technologies. Specifically, due to the conventional requirement for a thymine immediately upstream of the TALE target sequences (*i.e.*, the 5′-T constraint), over 150 loci in the human mitochondrial genome are presumed to be inaccessible to DdCBEs. Previous attempts at circumventing this requirement, either by developing monomeric DdCBEs or utilizing DNA-binding domains alternative to TALEs, have resulted in suboptimal specificity profiles with reduced therapeutic potential. Here, aiming to challenge and elucidate the relevance of the 5′-T constraint in the context of DdCBE-mediated mtDNA editing, and to expand the range of motifs that are editable by this technology, we generated DdCBEs containing TALE proteins engineered to recognize all 5′ bases. These modified DdCBEs are herein referred to as αDdCBEs. Notably, 5′-T-noncompliant canonical DdCBEs efficiently edited mtDNA at diverse loci. However, they were frequently outperformed by αDdCBEs, which exhibited significant improvements in activity and specificity, regardless of the most 5′ bases of their TALE binding sites. Furthermore, we showed that αDdCBEs are compatible with the enhanced DddA_tox_ variants DddA6 and DddA11, and we validated TALE shifting with αDdCBEs as an effective approach to optimize base editing outcomes. Overall, αDdCBEs enable efficient, specific, and unconstrained mitochondrial base editing.

## INTRODUCTION

Mitochondria are semiautonomous organelles with a central role in energy metabolism that contain a circular and multicopy genome, mitochondrial DNA (mtDNA), which in humans encodes 37 genes critical for oxidative phosphorylation.^1–7^ Pathogenic mtDNA variants are prevalent in ∼1 in 5,000 people and are causal in currently incurable metabolic disorders.^8–12^ Mitochondrial base editing has recently emerged as a potential therapeutic approach for these mtDNA-based diseases.^13–16^ Notably, given the challenging nature of exogenous RNA import into mitochondria, the effective manipulation of mtDNA is enabled by all-protein systems.^16–20^ In light of their rapid and accessible engineering, transcription activator-like effectors (TALEs) are the most commonly used DNA-binding domains in most current mitochondrial base editing technologies.^16,21–36^

DddA-derived cytosine base editors (DdCBEs), consisting of pairs of mitochondrially targeted fusion proteins composed of a TALE, one half of a split dsDNA deaminase toxin (DddA_tox_), and a uracil glycosylase inhibitor, represent the most extensively developed tools for mtDNA editing.^16,30–34^ Based on prior work on TALE-DNA interactions in native contexts and in gene targeting applications in the nuclear compartment,^23–27,37–47^ the target flexibility of DdCBEs on mtDNA is presumed to be constrained by the requirement of a 5′ thymine (5′-T) in their TALE binding sites, restricting over 150 loci in the human mitochondrial genome.^13,48^ This design guideline stems from the specific interaction between the highly conserved N-terminal domain (NTD) of wild-type TALEs and the 5′-T of their target sequences.^37–40^ Despite being generally followed, the significance of this constraint for the design of effective DdCBEs is yet to be characterized.^13,16,30–34^

Here, we sought to elucidate the relevance of the TALE 5′-T rule for DdCBE-mediated mitochondrial base editing, as well as to expand the targeting scope and design flexibility of this technology. To this end, building on our recently established system for the assembly of TALE-guided deaminases,^49–51^ we generated DdCBE variants that contain a previously developed TALE NTD engineered to accommodate any 5′ base.^52^ We designated these unconstrained DdCBEs as αDdCBEs. We conducted direct comparisons between DdCBEs and αDdCBEs in six mitochondrial genes, *ND4*, *ND2*, *ATP6*, *CO1*, *TC*, and *TL1*. Remarkably, we noted that breaking the 5′-T rule did not obligately preclude mtDNA editing with DdCBEs. However, αDdCBEs consistently outperformed canonical DdCBEs, thereby supporting unconstrained mtDNA editing as a potential strategy for disease modeling and gene therapy applications.

## MATERIALS AND METHODS

### Construction of FusX-compatible DdCBE backbone plasmids

All backbone plasmids were made via restriction cloning. A list including the source material for each construct is provided in Supplementary Table S1. In general, insert and vector bands were separated by agarose gel electrophoresis and purified with the Monarch DNA Gel Extraction Kit obtained from New England Biolabs (NEB). Ligations were done with the Quick Ligation^TM^ Kit (NEB). NEB^®^ Stable Competent *E. coli* (C3040H) were used for propagation, following the high efficiency transformation protocol specified by the manufacturer, and incubating plates and liquid cultures at 30°C. Plasmids were purified with the QIAprep Spin Miniprep Kit (Qiagen) and sequence-verified via whole-plasmid sequencing (Primordium Labs).

### Assembly of DdCBE-encoding plasmids

All DdCBE-encoding plasmids used in this study were assembled via the FusX TALE Base Editor (FusXTBE) platform.^49–51^ Briefly, following standard design rules,^13,53^ DdCBEs were designed *in silico* with TALE Writer^50,51^ and SnapGene. Specifically, TALE repeat arrays were designed to target between 15 and 17 bp, separated by spacers ranging from 11 to 18 bp long.^13,50,53^ A list of all TALE binding sites is provided in Supplementary Table S2. DdCBE-encoding plasmids were assembled via Golden Gate cloning.^49–51^ Primers used for colony PCR (see Supplementary Table S3) were synthesized as standard DNA oligos by Integrated DNA Technologies (IDT). NEB^®^ stable competent *E. coli* (C3040H) were used for propagation. Plasmids were purified with the QIAprep Spin Miniprep Kit (Qiagen) and sequence-verified via whole-plasmid sequencing (Primordium Labs).

### Generation of TALE-free constructs

Plasmids encoding TALE-free MTS–G1397-split DddA_tox_/DddA6/DddA11–UGI were generated with the Q5^®^ Site-Directed Mutagenesis (SDM) Kit (NEB), using the corresponding herein developed backbone plasmids as templates for each final construct containing the DddA_tox_, DddA6, or DddA11 C- or N-terminal halves.^13,53^ Cloning was carried out following the manufacturer’s instructions with the provided NEB^®^ 5-alpha competent *E. coli* cells. Primers for SDM (see Supplementary Table S3) were designed using NEBaseChanger (NEB) and synthesized as standard DNA oligos (IDT). Plasmids were purified with the QIAprep Spin Miniprep Kit (Qiagen) and sequence-verified via whole-plasmid sequencing (Primordium Labs).

### Mammalian cell culture and lipofection

HEK293T cells (CRL-3216^TM^, ATTC) were maintained at 37°C and 5% CO_2_. The cells were cultured in high-glucose DMEM (Thermo Fisher Scientific) supplemented with 10% (v/v) fetal bovine serum (Thermo Fisher Scientific) and 100 U ml^−1^ penicillin–streptomycin (Thermo Fisher Scientific). Lipofectamine^TM^ 3000 Transfection Reagent (Thermo Fisher Scientific) was used for lipofections. In brief, 24 h before lipofection, 0.3 × 10^6^ cells/well were seeded in 6-well plates. Then, lipofections proceeded with 500 ng per monomer for DdCBEs and TALE-free constructs to make up 1,000 ng of total plasmid DNA,^13,53^ and 1,000 ng of plasmid DNA for monomeric DdCBEs (mDdCBEs).^48^ Cells were collected for genotyping at 72 h post-transfection.

### Genomic DNA isolation from mammalian cell culture

At 72 h post-transfection, the cell medium was aspirated, the cells were washed with 500 µL 1× DPBS without calcium or magnesium (Thermo Fisher Scientific), trypsinized with 500 µL 1× Trypsin-EDTA (0.5%) without phenol red (Thermo Fisher Scientific) for 5 min at 37°C and collected in microcentrifuge. Total genomic DNA (including mitochondrial DNA) was purified using the DNeasy Blood & Tissue Kit (Qiagen) following the manufacturer’s instructions and stored at −20°C until further downstream processing.

### High-throughput sequencing of genomic DNA samples

Genotyping primers were designed using Primer-BLAST,^54^ querying the *Homo sapiens* genome assembly hg38 for primer pair specificity. In detail, to increase PCR specificity for the intended mitochondrial target sequences, PCR was biased against the amplification of nuclear mitochondrial pseudogenes (NUMTs)^55^ by aligning the 3′ ends of candidate primers with specific single-nucleotide mismatches between intended mitochondrial targets and potential unintended nuclear templates, and accordingly adding 3′-terminal phosphorothioate (PS) bonds to the primers. This strategy avoids 3′-terminal editing of the mismatched primers by the 3′−5′ exonuclease activity of Q5^®^ High-Fidelity DNA Polymerase (NEB), increasing PCR specificity.^56^

Primers including the partial Illumina^®^ forward and reverse adapter sequences, in addition to barcodes for sample multiplexing, were synthesized as Ultramer^TM^ DNA oligos (IDT). Afterward, genomic sites of interest were amplified with the Q5^®^ High-Fidelity 2X Master Mix (NEB) using conventional thermocycling conditions. Then, PCR products corresponding to the same experimental condition but with different barcodes were combined after agarose gel electrophoresis, purified using the Monarch DNA Gel Extraction Kit (NEB), confirmed via Sanger sequencing (Genewiz), and submitted to next-generation sequencing (NGS, Amplicon-EZ with partial adapters, Genewiz). Alternatively, if the samples were not multiplexed, the PCR products were individually purified with the QIAquick PCR Purification Kit (QIAGEN), confirmed via Sanger sequencing (Genewiz), and submitted to NGS (Amplicon-EZ without partial adapters, Genewiz). A list of all genotyping primers is provided in Supplementary Table S4.

### Analysis of high-throughput sequencing data

In multiplexed samples, the paired-end read FASTQ files generated by NGS were demultiplexed and analyzed utilizing the CRISPRessoPooled tool within CRISPResso2.^57,58^ Similarly, if the samples were not multiplexed, the paired-end read FASTQ files were analyzed with the CRISPRessoBatch tool within CRISPResso2.^57,58^ In general, DdCBE spacer sequences were used as the guide sequence input. Besides, for each replicate in each experimental condition, the sequence of the amplicon corresponding to the target site, plus the respective barcode if demultiplexing, was used as the amplicon sequence input. Unless otherwise stated, the quantification window size was set to 8 or 10, and the quantification window center was set to −8 or −10. All optional parameters were set to NA.^13,57^

The output allele frequency table was used to determine the overall on-target editing in each sample, calculated as the percentage of aligned reads with C•G-to-T•A conversions within a spacer.^48^ Likewise, the output nucleotide percentage table was used to calculate the editing activity at each cytosine within each spacer, as well as the proximal off-target editing within each amplicon.^29,48^ In detail, similar to the methodology followed in the development of zinc-finger DdCBEs (ZF-DdCBEs), average amplicon-wide off-target editing was quantified as the sum of all C•G-to-T•A conversions within an amplicon, excluding its corresponding DdCBE spacer, over the total number of C•G base pairs within that amplicon.^29^ Calculations were done in Microsoft Excel.

### Targeted amplicon sequencing for nuclear DNA off-target analyses

Based on previous reports, nested PCR was performed to amplify a TALE-dependent off-target site within the NUMT *MTND4P12*, and conventional PCR to amplify a frequent TALE-independent off-target site at chr8:37153384C (hg38).^13,59,60^ Primers for the first PCR (PCR1) in the nested PCR strategy were synthesized as standard DNA oligos (IDT). Primers for the generation of amplicons for NGS were synthesized as Ultramer^TM^ DNA oligos (IDT), including barcodes and Illumina^®^ adapters. PCR was done with the Q5^®^ High-Fidelity 2X Master Mix (NEB). After PCR1 in the nested PCR strategy, amplicons were purified with the QIAquick PCR Purification Kit (Qiagen), and 10 ng were used as template DNA for the second PCR. Amplicons for targeted deep sequencing were purified as detailed in the section “High-Throughput Sequencing of Genomic DNA Samples”” and submitted to NGS (Amplicon-EZ with partial adapters, Genewiz).

The methodology for data analysis was similar to the approach described in the section “Analysis of High-Throughput Sequencing Data.” In detail, to determine the overall nuclear DNA off-target editing at *MTND4P12*, the CRISPResso2 output allele frequency table was used to calculate the percentage of aligned reads with C•G-to-T•A conversions within the pseudospacer (*i.e.*, the nuclear DNA region analogous to the genuine target spacer in mtDNA). In addition, the output nucleotide percentage table was used to calculate the editing activity at each cytosine within the pseudospacer. Similarly, the nucleotide percentage table was used to quantify the nuclear DNA off-target editing at the abovementioned TALE-independent off-target locus.

### Sanger sequencing of genomic DNA samples and data analysis

For the 5′ nucleotide precedence analyses at the *ATP6* locus, genomic DNA from *ATP6*-edited cells and the corresponding controls was purified as described in the section “Genomic DNA Isolation from Mammalian Cell Culture.” Afterward, PCR was conducted with the Q5^®^ 2X Master Mix (NEB) and *ATP6* Sanger sequencing primers (listed in Supplementary Table S4), which were designed as detailed in the section “High-Throughput Sequencing of Genomic DNA Samples” (without adapters or barcodes) and synthesized as standard DNA oligos with 3′ PS bonds (IDT). Then, PCR products were visualized by electrophoresis in a 1% agarose gel, purified with the QIAquick PCR Purification Kit (QIAGEN), and submitted to Sanger sequencing (Genewiz). The resulting trace (.ab1) files were analyzed in the EditR server.^61^ Briefly, DdCBE spacer sequences were used as the guide sequence input.^50^ In addition, the 5′ starts and 3′ ends of the trace files were trimmed to exclude bases with quality scores lower than 40, and the *p* value cutoff for calling base editing was set to 0.01. Besides, to exclude noise from low-confidence measurements in the calculation of the average editing efficiencies, these were computed per replicate as the mean of the predicted editing at cytosines within the spacer that corresponded to highly significant (*p* ≤ 0.01) editing events, compared with untreated controls. Statistical analyses were conducted using two-tailed unpaired *t* tests in GraphPad Prism 10.

## RESULTS

### Mitochondrial base editing with αDdCBEs

**Figure 1.**
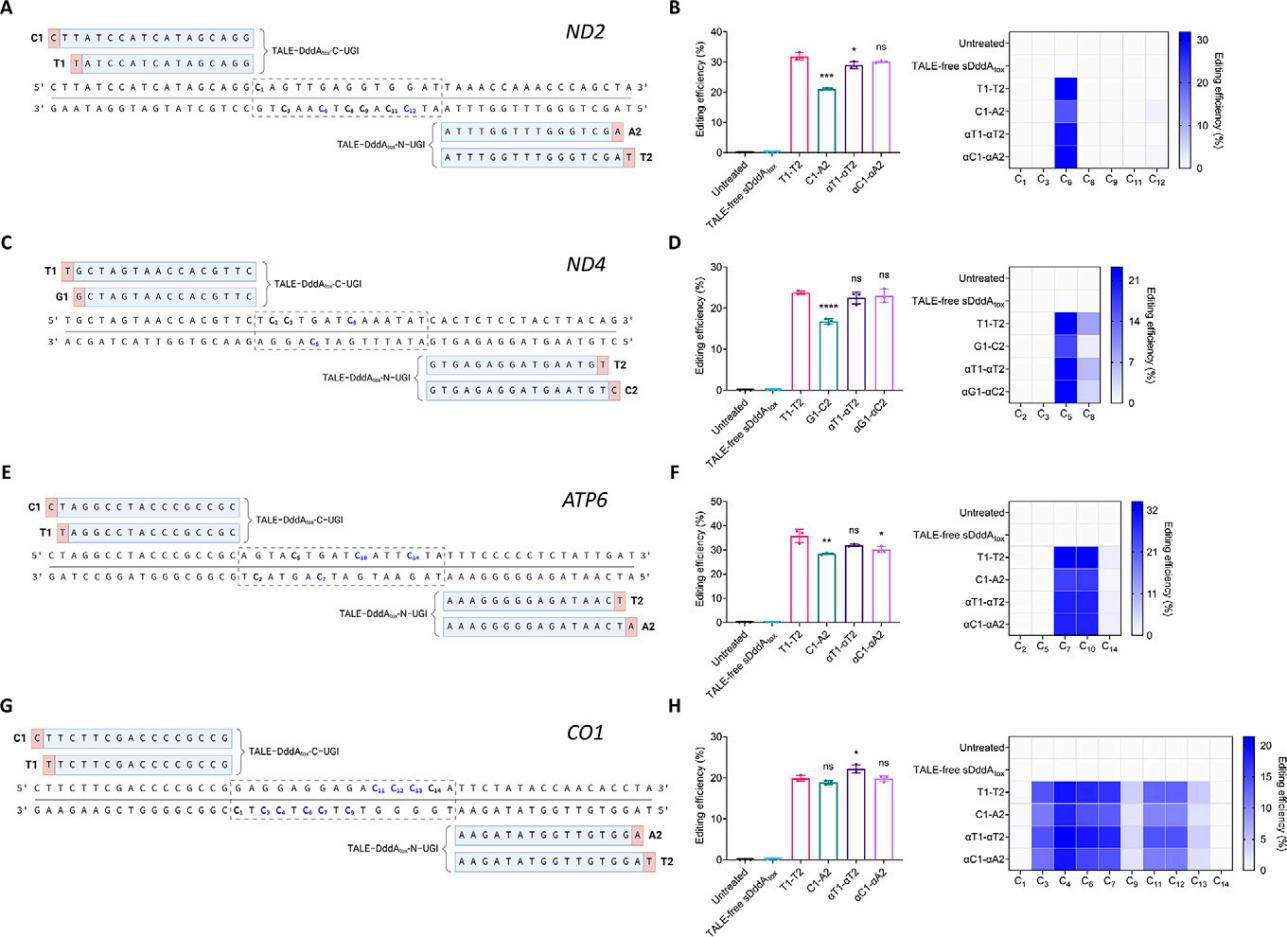
Mitochondrial base editing with 5′-T-compliant and 5′-T-noncompliant DdCBEs and αDdCBEs. **(A)**, **(C)**, **(E)**, **(G)** Target sites within *ND2*, *ND4*, *ATP6*, and *CO1*, respectively. The sequences targeted by the TALE repeat arrays are shown in the blue rectangles, and the nucleotides immediately upstream of these sequences are indicated in the red boxes. Each base editor arm is denominated as N1/N2 (where ‘N’ represents A, C, G, or T) depending on the corresponding most 5′ nucleotide of its TALE binding site and whether it constitutes the left (‘1’) or the right (‘2’) arm of the construct. The spacers (sequences between TALE target sequences, where base editing is expected) are enclosed in dashed rectangles. All cytosines within the spacers are in bold and numbered relative to their positions from the 3′ end of their respective left arms. Cytosines consistently edited at ≥1% across experimental conditions are highlighted in blue. Created with BioRender.com. **(B)**, **(D)**, **(F)**, **(H)** Overall editing efficiencies (left) and corresponding editing patterns (right) induced by 5′-T-compliant and 5′-T-noncompliant DdCBEs and αDdCBEs at *ND2*, *ND4*, *ATP6*, and *CO1*, in that order. TALE-free sDddA_tox_: N- and C-termini of TALE-free, mitochondrially targeted, split DddA_tox_–UGI. T1-T2: 5′-T-compliant DdCBE pairs. V1-V2 (where ‘V’ represents a non-T nucleotide): 5′-T-noncompliant DdCBE pairs. αT1-αT2: 5′-T-compliant αDdCBE pairs. αV1-αV2: 5′-T-noncompliant αDdCBE pairs. All measurements were obtained via NGS and correspond to editing efficiencies in HEK293T cells 3 days post-transfection. Values and error bars in **(B)**, **(D)**, **(F)**, and **(H)** represent the mean ± SD of *n* = 3 independent biological replicates. Displayed statistical significances were determined by comparing against the respective T1-T2 condition. *p < 0.05; **p < 0.01; ***p < 0.001; ****p < 0.0001; ns (not significant), *p* > 0.05 by two-tailed unpaired *t test* in GraphPad Prism 10. TALE, transcription activator-like effector; NGS, next-generation sequencing.

In general, most TALEs require a thymine base immediately upstream of their target sequences for efficient TALE-DNA binding.^37–40^ Thus, canonical DdCBEs, which contain standard TALE proteins, are predicted to induce efficient mtDNA editing only in 5′-T-compliant formats, that is, when the TALE target sequences are preceded by a thymine.^13,48^ Consequently, given the ability of the modified TALE NTD to recognize all 5′ bases,^52^ we hypothesized that αDdCBEs can edit mtDNA as efficiently as standard, 5′-T-compliant DdCBEs, regardless of the TALE 5′-T rule. Moreover, we expected 5′-T-noncompliant DdCBEs to induce poor editing efficiencies relative to 5′-T-compliant DdCBEs. To test these hypotheses, we compared pairs of DdCBEs and αDdCBEs in both 5′-T-compliant and 5′-T-noncompliant formats at four mitochondrial loci in HEK293T cells (Fig. 1). To avoid variations in base editing outcomes as a result of spacer variability, we maintained fixed spacer sequences at each target locus by lengthening or shortening the TALE binding sites from the 5′ ends by a maximum of 2 bp each.

In addition to the untreated condition, TALE-free MTS–split DddA_tox_–UGI (labeled as TALE-free sDddA_tox_) was used as a negative control. Additionally, DdCBE pairs are generally referred to as N1-N2 (N = A, C, G, or T), denoting the most 5′ base of either the left (1) or the right (2) TALE. Accordingly, αDdCBEs are designated as αN1-αN2. Hence, T1-T2 denotes a 5′-T-compliant DdCBE pair, which corresponds to a positive control. Besides, unless otherwise noted, all base editors were designed with G1397-split DddA_tox_ in the C-to-N configuration, that is, left TALE–DddA_tox_-C–UGI + right TALE–DddA_tox_-N–UGI.^13^

Based on previous work on the development of DdCBEs and the FusXTBE platform, the mitochondrial genes *ND2*, *ND4*, and *CO1* were chosen for these experiments.^13,50^ In addition, the target site within the *ATP6* locus was selected due to its sequence structure, which enabled the testing of several combinations of base editors with TALEs preceded by any 5′ base (explored in detail in Fig. 3).

At the *ND2* locus, we observed that C1-A2 was the least active base editor, reaching overall editing efficiencies of ∼21%, whereas T1-T2, αT1-αT2, and αC1-αA2 displayed editing frequencies ranging from ∼29% to ∼32%. In addition, all *ND2* base editors resulted in similar mutation patterns (Fig. 1A, B). Similarly, at the *ND4* site, we found that G1-C2 induced editing efficiencies of ∼17%, the lowest compared with T1-T2, αT1-αT2, and αG1-αC2, which installed edits at frequencies between ∼22% and ∼24%. Interestingly, both G1-C2 and αG1-αC2 resulted in more specific mutation patterns within the spacer than their 5′-T-compliant counterparts (Fig. 1C, D). Comparably, at the *ATP6* locus, C1-A2 was less efficient than T1-T2, with editing frequencies of ∼28% compared with ∼36%, respectively. Moreover, both αT1-αT2 and αC1-αA2, which displayed editing frequencies of up to ∼32%, were nearly as effective as T1-T2. Notably, all *ATP6* base editors displayed similar editing patterns (Fig. 1E, F). Unexpectedly, at the *CO1* site, all base editors displayed similar levels of activity and mutation patterns, with overall efficiencies ranging from ∼19 to ∼22%. Besides, despite the preference of DddA_tox_ for cytosines in TC motifs,^13^ several non-TC motifs within the *CO1* spacer were efficiently edited (Fig. 1G, H).

Collectively, these results suggest that canonical DdCBEs can effectively edit mtDNA even if their respective TALEs break the 5′-T rule. However, αDdCBEs tend to perform similarly to 5′-T-compliant DdCBEs and outperform 5′-T-noncompliant DdCBEs, thereby surpassing canonical DdCBEs in regard to design flexibility.

### Characterizing off-target editing by αDdCBEs

Seeking to characterize the specificity profiles of αDdCBEs relative to DdCBEs, based on an approach reported by Willis et al.,^29^ we calculated the normalized ratios between the on-target (*i.e.,* within the spacer) and average amplicon-wide off-target editing efficiencies for each base editor. These quantities enabled us to conduct direct comparisons between the overall performance of αDdCBEs in contrast to DdCBEs, both in terms of their on-target editing activities and proximal off-target effects.

**Figure 2.**
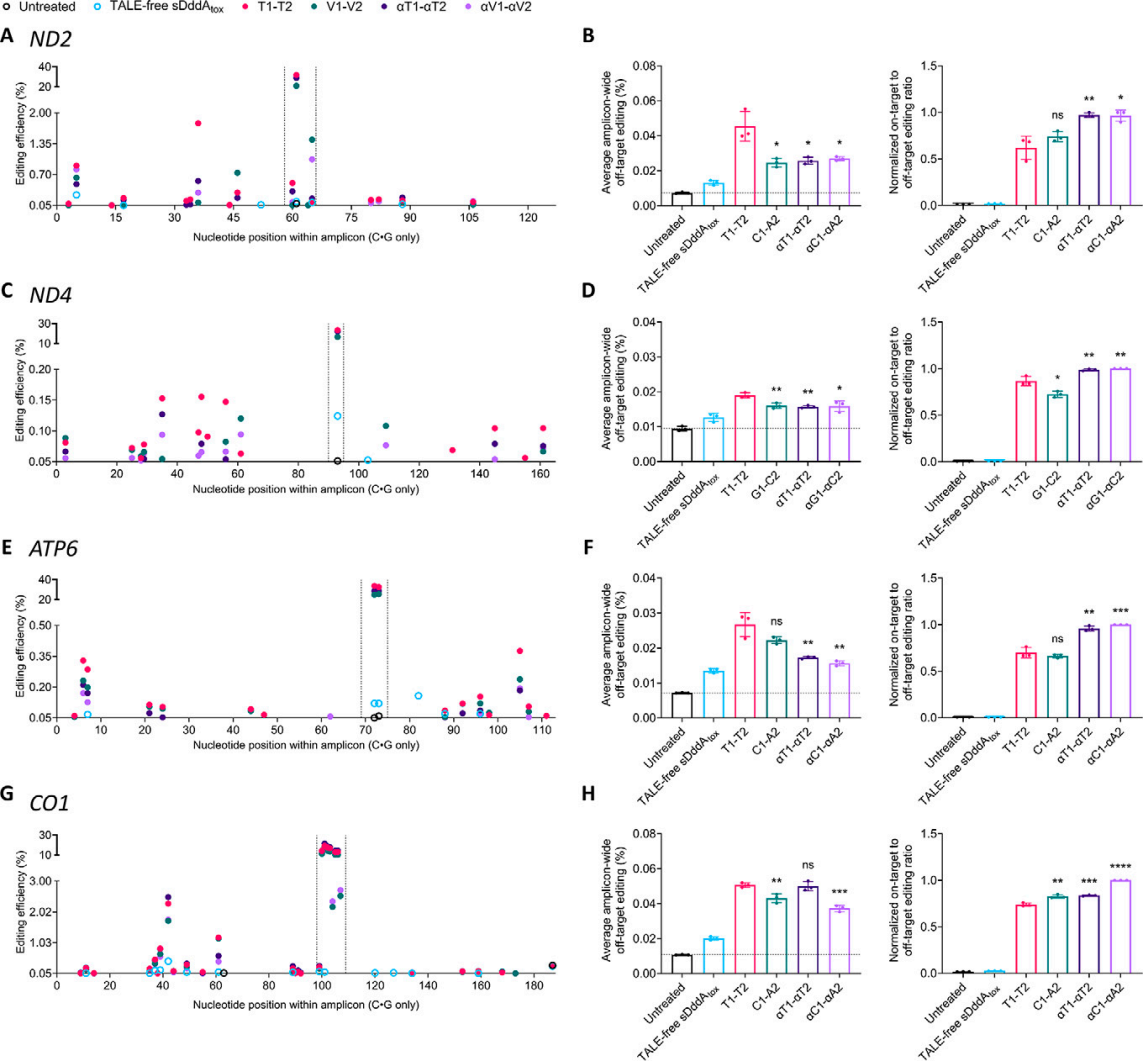
Proximal off-target effects by 5′-T-compliant and 5′-T-noncompliant DdCBEs and αDdCBEs. **(A)**, **(C)**, **(E)**, **(G)** Amplicon-wide editing efficiencies induced by 5′-T-compliant and 5′-T-noncompliant DdCBEs and αDdCBEs at *ND2*, *ND4*, *ATP6*, and *CO1*, respectively. The narrow regions between the vertical dashed lines confine the cytosines within the spacers at each target site. The vertical axes are divided into two segments, with the ranges of the bottom segments adjusted to showcase the amplitudes of the proximal off-target effects, and the ranges of the top segments adjusted to showcase the amplitudes of the on-target editing events. All measurements were obtained via NGS and correspond to editing efficiencies in HEK293T cells 3 days post-transfection. All values represent the mean of *n* = 3 independent biological replicates. TALE-free sDddA_tox_: N- and C-termini of TALE-free, mitochondrially targeted, split DddA_tox_–UGI. T1-T2: 5′-T-compliant DdCBE pairs. V1-V2 (where ‘V’ represents a non-T nucleotide): 5’-T-noncompliant DdCBE pairs. αT1-αT2: 5′-T-compliant αDdCBE pairs. αV1-αV2: 5’-T-noncompliant αDdCBE pairs. **(B)**, **(D)**, **(F)**, **(H)** Average amplicon-wide off-target editing (left) and normalized on-target-to-off-target editing ratios (right) of 5′-T-compliant and 5’-T-noncompliand DdCBEs and αDdCBEs at *ND2*, *ND4*, *ATP6*, and *CO1*, in that order. The horizontal dashed line in the average amplicon-wide off-target editing bar graphs corresponds to the mean of the untreated condition. Values and error bars in **(B)**, **(D)**, **(F)**, and **(H)** represent the mean ± SD of *n* = 3 independent biological replicates. Displayed statistical significances were determined by comparing against the respective T1-T2 condition. *p < 0.05; **p < 0.01; ***p < 0.001; ****p < 0.0001; ns (not significant), *p* > 0.05 by two-tailed unpaired t test in GraphPad Prism 10.

At the *ND2* locus, C1-A2, αT1-αT2, and αC1-αA2 resulted in an ∼1.8-fold reduction in average amplicon-wide off-target editing compared with T1-T2 (Fig. 2A). Accordingly, given their high on-target editing activities (Fig. 1B) and relatively low off-target effects (Fig. 2B, left), αDdCBEs considerably outperformed their canonical counterparts at the *ND2* site (Fig. 2B, right). In contrast, all *ND4* base editors introduced off-target editing at frequencies below 0.2% throughout the amplicon (Fig. 2C). However, given the relatively low on-target activity displayed by G1-C2 (Fig. 1D), and the moderately higher off-target effects caused by T1-T2 compared with the other pairs (Fig. 2D, left), at the *ND4* locus, αDdCBEs outperformed DdCBEs (Fig. 2D, right). Similarly, all *ATP6* base editors introduced off-target cytosine conversions at rates below 0.5% (Fig. 2E). However, both T1-T2 and C1-A2 resulted in somewhat higher average amplicon-wide off-target editing than αT1-αT2 and αC1-αA2 (Fig. 2F, left). Consequently, at the *ATP6* site, αDdCBEs performed better than DdCBEs (Fig. 2F, right). In contrast, at *CO1*, the 5′-T-compliant pairs resulted in higher average amplicon-wide off-target editing efficiencies compared with the 5′-T-noncompliant pairs (Fig. 2G and Fig. 2H, left). Therefore, both 5′-T-noncompliant *CO1* base editors outperformed the 5′-T-compliant DdCBE pair, T1-T2, with αC1-αA2 showing the highest overall performance (Fig. 2H, right).

Subsequently, to further characterize the specificity profiles of αDdCBEs relative to DdCBEs, we investigated their nuclear off-target effects at a TALE-dependent site (*MTND4P12*) and a TALE-independent site (chr8:37153384C, hg38) in *ND4*-edited cells (Supplementary Fig. S1).^13,60^ Notably, the *MTND4P12* off-target and the *ND4* on-target sequences differ by a single G/A mismatch (Supplementary Fig. S1A). Remarkably, at *MTND4P12*, T1-T2 resulted in off-target editing efficiencies of ∼16%. In contrast, all other pairs achieved frequencies of ∼0.2% (G1-C2), ∼7.5% (αT1-αT2), and ∼1% (αG1-αC2) (Supplementary Fig. S1B,C). On the contrary, at the herein examined TALE-independent off-target region, which was previously identified by Lei et al. as a frequently observed nuclear off-target across DdCBEs, and shares no sequence homology with the *ND4* on-target sequence, the editing efficiencies remained substantially similar among base editors. Nonetheless, αG1-αC2 resulted in moderately higher cytosine conversion rates relative to all other editors, although at efficiencies below 0.2%. Besides, TALE-free sDddA_tox_ led to off-target editing with frequencies of ∼1% (Supplementary Fig. S1D).

As a whole, these results suggest that, in the scope of proximal off-target effects in mtDNA and, potentially, nuclear editing at TALE-dependent off-target sites, αDdCBEs tend to be more specific than standard, 5′-T-compliant DdCBEs, thereby outperforming them in terms of specificity.

### Comparative analyses of the on-target activities of DdCBEs and αDdCBEs preceded by all 5′ bases

**Figure 3.**
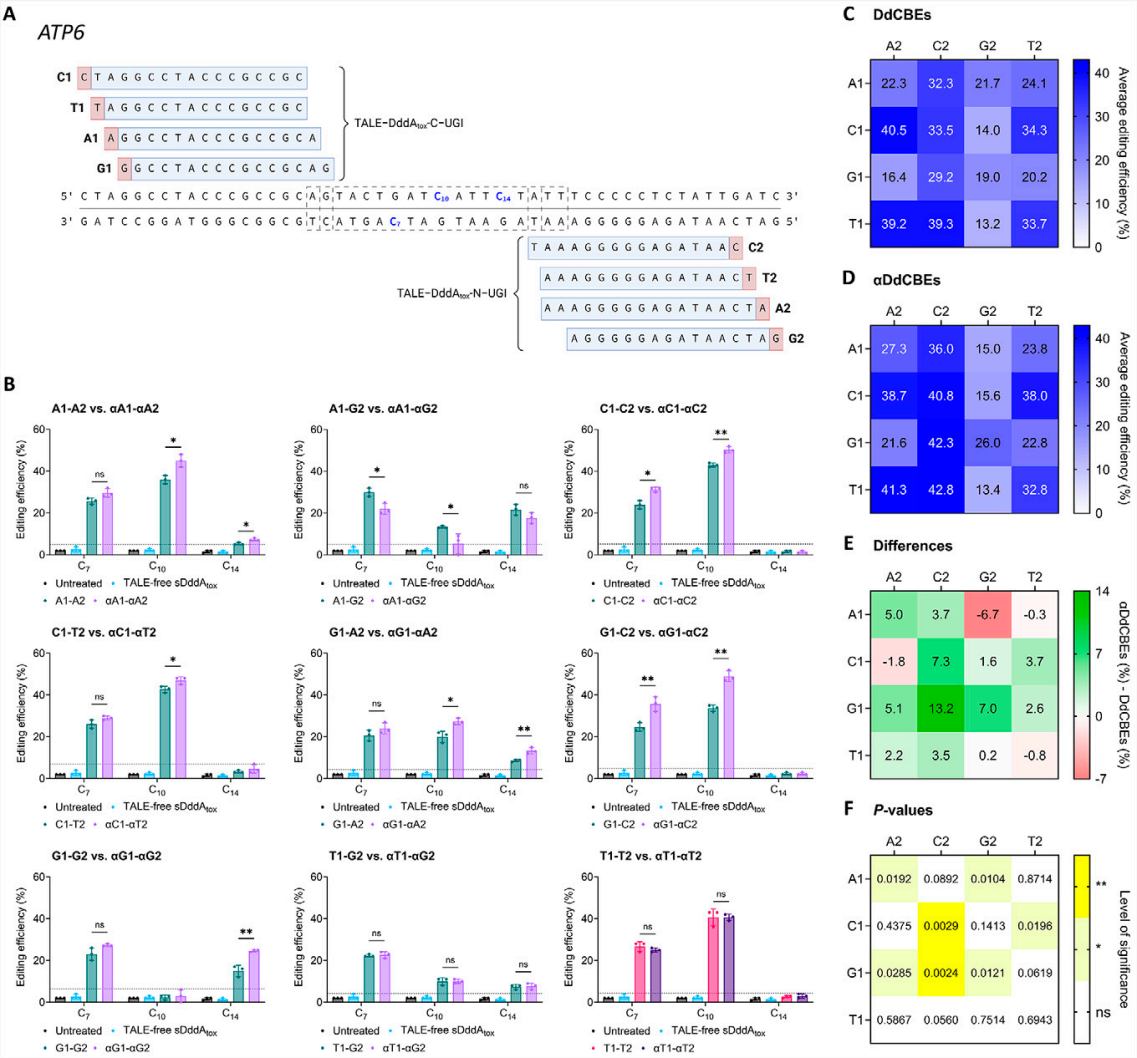
On-target editing efficiencies at *ATP6* with DdCBEs and αDdCBEs preceded by all 5′ bases. **(A)** Target site within *ATP6*. The spacers are enclosed in dashed rectangles. The cytosines that were consistently edited across conditions are highlighted in bold blue and numbered from the 3′ end of the T1 arm. Created with BioRender.com. **(B)** Representative comparisons between the editing efficiencies induced by DdCBE and αDdCBE pairs at *ATP6*. TALE-free sDddA_tox_: N- and C-termini of TALE-free, mitochondrially targeted, split DddA_tox_–UGI. N1-N2 (where ‘N’ represents A, C, G, or T): canonical DdCBE pairs. αN1-αN2: αDdCBE pairs. T1-T2 and αT1-αT2 are shown in red and purple, respectively, to maintain the color scheme used in previous figures. All measurements were obtained via Sanger sequencing and correspond to editing efficiencies in HEK293T cells 3 days post-transfection. Values and error bars represent the mean ± SD of *n* = 3 independent biological replicates. The horizontal dashed lines correspond to critical percent values, obtained from sequencing trace decomposition analyses with a *p* value cutoff of 0.01, above which base editing estimates are significantly different from background. **(C)**, **(D)** Average DdCBE- and αDdCBE-induced editing efficiencies at each specified condition. **(e)** Differences between the average editing efficiencies induced by αDdCBEs and DdCBEs. **(f)** Corresponding *p* values for statistical comparisons between the average editing efficiencies displayed in **(c)** and **(d)**. *p < 0.05; **p < 0.01; ns (not significant), *p* > 0.05 by two-tailed unpaired t test in GraphPad Prism 10.

We then explored whether αDdCBEs consistently led to on-target (within a spacer) base editing enhancements relative to DdCBEs, regardless of the 5′ bases of their TALE binding sites. To this end, we identified *ATP6* as a locus accessible by base editors containing TALEs targeting sequences preceded by A, C, G, or T, with moderate variability across the resulting spacers. We designed TALE proteins containing between 15 and 17 repeats, and delimiting spacers ranging from 13 to 18 bp long. Of note, all spacers contained a bottom- and a top-strand cytosine approximately halfway through, and an additional top-strand cytosine toward the 3′ end (Fig. 3A).

We observed that equivalent base editors (*i.e.,* A1-A2 and αA1-αA2, A1-C2 and αA1-αC2, and so on) led to similar mutation patterns, although frequently with moderately different efficiencies. Representative comparisons between equivalent pairs are shown in Figure 3B, and the complete set is displayed in Supplementary Figure S2. Afterward, we calculated the average activities of each construct to elucidate the general differences between αDdCBE- and DdCBE-induced base editing frequencies at *ATP6* (Fig. 3C, D). Then, we determined the differences between these quantities for each pair of equivalent base editors, along with their respective significance levels, facilitating the visualization of the overall αDdCBE-induced activity enhancements across comparisons (Fig. 3E, F). Notably, broad improvements in the efficiency of base editing with αDdCBEs relative to DdCBEs were observed in 6 out of the 16 total comparisons (Fig. 3C–F).

It is worth noting that *ATP6* base editors containing G2/αG2 arms were generally the least effective across conditions (Fig. 3C, D); moreover, only αA1-αG2 led to a statistically significant overall activity reduction relative to its canonical counterpart (Fig. 3E, F). Furthermore, only G2/αG2-containing pairs, except for G1-G2 and αG1-αG2, targeted spacer sequences with lengths of 17 or 18 bp (Fig. 3A). DddA-derived base editors targeting spacers of such lengths often install lower editing efficiencies compared with pairs with spacers up to 16 bp long.^13,53^ Thus, we hypothesized that reducing the spacers of G2/αG2-containing pairs to 16 bp or less would improve their editing efficiencies.

To test this hypothesis and characterize the effects of decreasing spacer length on the activities of A1-G2 versus αA1-αG2, we evaluated two additional sets of arms: G2.16/αG2.16 and G2.17/αG2.17 (Supplementary Fig. S3A). Interestingly, G2.16/αG2.16-containing pairs led to lower editing efficiencies relative to the initial G2/αG2-containing pairs (designated as G2.15/αG2.15 in Supplementary Fig. S3), as well as minimal αDdCBE-induced enhancements. Strikingly, G2.17/αG2.17-containing pairs, with shorter spacers, led to improvements in base editing efficiencies relative to the original pairs, as well as greater αDdCBE-induced base editing reductions (Supplementary Fig. S3B).

Overall, these results further indicate that DdCBEs and αDdCBEs can effectively edit mtDNA in 5′-T-noncompliant formats. However, αDdCBEs can lead to greater mtDNA editing efficiencies than their canonical counterparts, although in particular contexts the opposite can be observed.

### αDdCBEs outperform DdCBEs at mtDNA sites with stretches without 5′-T nucleotides

**Figure 4.**
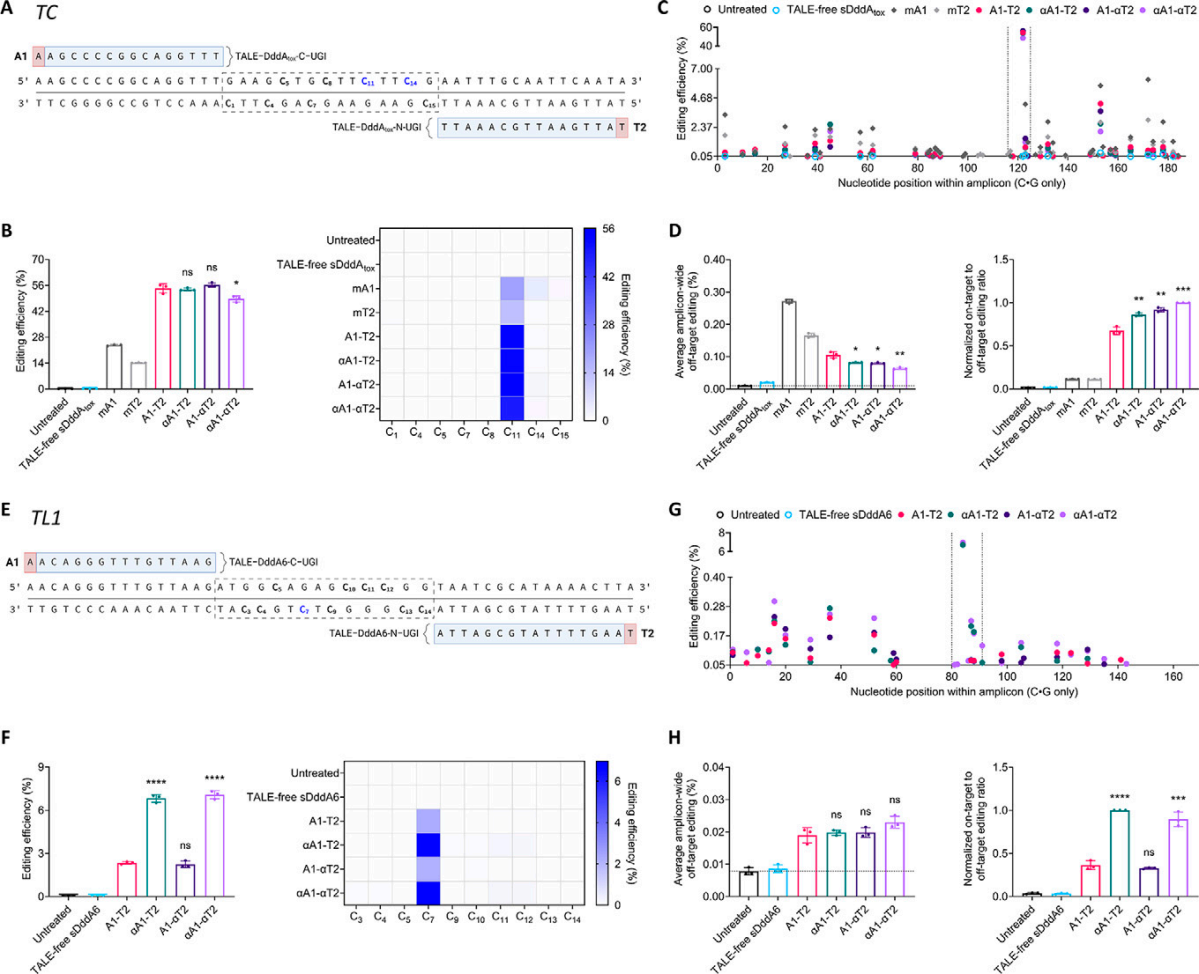
αDdCBEs effectively edit mtDNA at sites with stretches without 5′-T nucleotides. **(A)**, **(E)** Target sites within the mitochondrial tRNA-Cys-encoding gene *TC* and the mitochondrial tRNA-Leu-encoding gene *TL1*, respectively. **(B)**, **(F)** Overall editing efficiencies (left) and corresponding editing patterns (right). Created with BioRender.com. **(C)**, **(G)** Amplicon-wide editing efficiencies. The narrow regions between the vertical dashed lines confine the on-target cytosines within the spacers. The vertical axes are divided into two segments, with the ranges of the bottom segments adjusted to showcase the amplitudes of the proximal off-target effects, and the ranges of the top segments adjusted to showcase the amplitudes of the on-target editing events. **(D)**, **(H)** Average amplicon-wide off-target editing (left) and normalized on-target-to-off-target editing ratios (right). The horizontal dashed line in the average amplicon-wide off-target editing bar graphs corresponds to the mean of the untreated condition. TALE-free sDddA_tox_/sDddA6: N- and C-termini of TALE-free, mitochondrially targeted, split DddA_tox_/DddA6–UGI. mA1: 5′-T-noncompliant monomeric DdCBE (mDdCBE) control. mT2: 5′-T-compliant mDdCBE control. A1-T2: canonical DdCBE pair. αA1-T2 and A1-αT2: partially modified pairs. αA1-αT2: αDdCBE pair. All measurements were obtained via NGS and correspond to editing efficiencies in HEK293T cells 3 days post-transfection. Values and error bars in (B)–(D) and (F)–(H) represent the mean ± SD of *n* = 3 independent biological replicates. Displayed statistical significances were determined by comparing against the respective A1-T2 conditions. *p < 0.05; **p < 0.01; ***p < 0.001; ****p < 0.0001; ns (not significant), *p* > 0.05 by two-tailed unpaired t test in GraphPad Prism 10.

Subsequently, we evaluated the effectiveness of αDdCBEs at target sites that, based on standard design principles,^13^ cannot be accessed without breaking the TALE 5′-T rule. To this purpose, we first assessed an array of base editors at the tRNA-Cys-encoding gene *TC*. This locus is reportedly editable by mDdCBEs but not by dimeric DdCBEs.^48^ In detail, we tested a standard pair (A1-T2), two partially modified pairs (αA1-T2 and A1-αT2), and a fully modified pair (αA1-αT2). In addition, we included two monomeric controls: mA1 and mT2, analogous to the left and right arms of the dimeric constructs (Fig. 4A). Unexpectedly, the dimeric base editors induced overall editing efficiencies ranging from ∼49% to ∼56%, well above mA1 (∼24%) and mT2 (∼14%) (Fig. 4B). Furthermore, A1-T2, αA1-T2, A1-αT2, and αA1-αT2 were considerably more specific than mA1 and mT2, which installed edits outside of the intended target sequence at frequencies of up to ∼6% and ∼3%, in that order (Fig. 4C and Fig. 4D, left). Therefore, the dimeric base editors far outperformed their monomeric counterparts, with αA1-αT2 exhibiting the greatest overall performance (Fig. 4D, right).

To further evaluate the effectiveness of αDdCBEs at target sites lacking accessible 5′-Ts, we assessed a set of base editors at the tRNA-Leu-encoding gene *TL1*. Pathogenic variants in this gene, such as the broadly prevalent m.3243A>G, are linked to impaired oxidative phosphorylation and a wide range of complex disease outcomes.^62–64^ We initially observed that DddA_tox_ was poorly active at *TL1*; hence, to obtain an ample range of activities for comparison purposes, we used DddA6, an enhanced variant of DddA_tox_, which showed improved editing efficiencies at this site (Supplementary Fig. S4). Of note, the denominations of the *TL1* base editors are similar to those of the *TC* pairs (Fig. 4E). Besides, given that monomeric variants for DddA6 are yet to developed,^48,53^ *TL1*-specific monomeric controls were not included. Remarkably, the αA1-containing pairs led to an approximately threefold increase in activity relative to A1-T2 and A1-αT2 (Fig. 4F). Moreover, all base editors resulted in similar specificity profiles; thus, the αA1-containing pairs significantly outperformed their A1-containing counterparts (Fig. 4H).

These results collectively suggest that dimeric DddA-derived base editors containing either canonical or unconstrained TALE NTDs, or both, can effectively access loci with stretches without 5′-Ts. Nevertheless, in some contexts, utilizing unconstrained TALEs to target sequences preceded by non-T nucleotides can facilitate the introduction of targeted modifications in mtDNA.

### TALE shifting with αDdCBEs as a strategy to fine-tune mitochondrial base editing outcomes

The outcomes of DddA-derived base editors are partly determined by spacer length and the positions of the target cytosines within the spacer.^13,28,29,53^ Given that these determinants are contributed by the DNA-binding domains, targeting a particular locus with different pairs of TALEs can lead to diverse editing outcomes.^32,65^ Indeed, depending on their TALE proteins, the *ATP6* base editors developed in this study resulted in distinct mutation patterns (Fig. 3B). Thus, focusing on the disease-relevant gene *TL1* and based on its optimized TALE formats (Fig. 4E–H), we aimed to explore TALE shifting with αDdCBEs as a strategy to fine-tune mitochondrial base editing outcomes.

Seeking to further increase the editing efficiencies at *TL1*, and considering that DddA6-containing base editors can result in modest levels of activity (Fig. 4F), we switched to DddA11, a deaminase variant with higher relative editing efficiencies compared with both DddA_tox_ and DddA6.^53^ Importantly, the enhanced activity of DddA11 can lead to decreased target selectivity, since it can process cytosines in non-TC motifs, which tend to be poorly processed by DddA_tox_ or DddA6.^53^ Nonetheless, for precision genome editing applications, we reasoned that the unconstrained TALEs of αDdCBEs could be shifted around a target site to mitigate unintended editing events within a spacer.

To investigate this premise, we focused on the m.3242G>A variant at the *TL1* locus, which is associated with various disease phenotypes, including mitochondrial myopathy.^66–73^ This mutation has been reported in patient tissues in both heteroplasmic (where wild-type and mutant mtDNA coexist) and homoplasmic states (where all mtDNA molecules contain the mutation). As a proof-of-concept, we attempted to install this point mutation at heteroplasmic levels *in vitro*. Notably, only a homoplasmic cellular model of the m.3242G>A variant has been developed to date, limiting the investigation of the heteroplasmic condition primarily to clinical observations and the analysis of patient tissues.^66–70^

**Figure 5.**
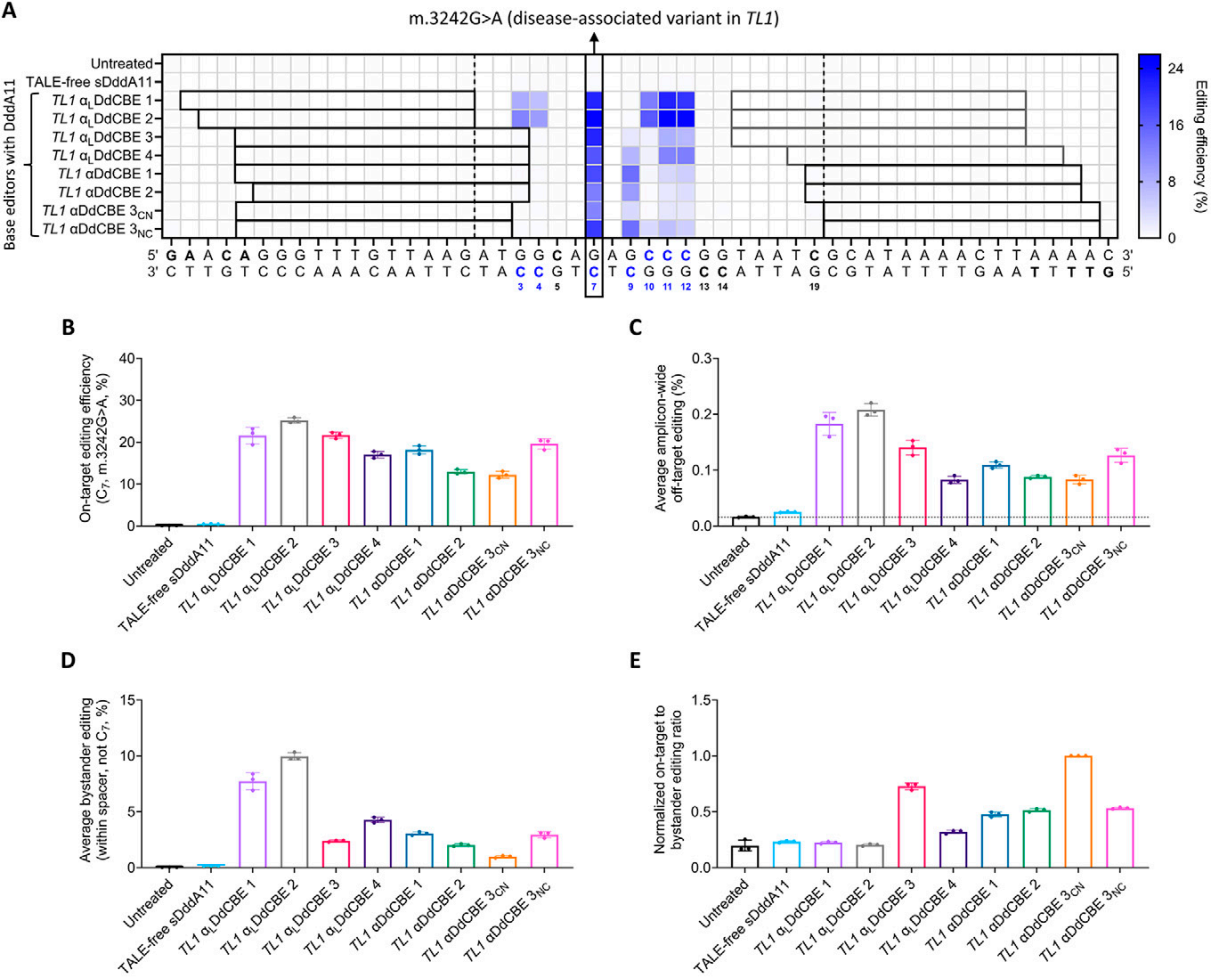
Fine-tuning mitochondrial base editing outcomes at the *TL1* locus via TALE shifting. **(A)** Heatmap detailing the editing patterns induced by DddA11-containing αDdCBEs and α_L_DdCBEs at *TL1*. The position of the m.3242G>A variant is highlighted in the vertical black rectangle. The TALE target sequences (excluding their 5′ nucleotides) are indicated in the horizontal black or dark gray rectangles, which correspond to unconstrained or canonical TALEs, respectively. α_L_DdCBEs are base editors where only the left (L) arm contains an unconstrained TALE. The vertical dashed lines define the overall spacer region, indicating the 3′ end of the leftmost TALE target sequence and the 5′ end of the rightmost TALE target sequence. The 5′ nucleotides of the TALE binding sites are shown in bold in the sequence below the heatmap. Cytosines within the overall spacer region are in bold and numbered relative to their positions from the left vertical dashed line. Cytosines that were edited at ≥1% are highlighted in blue. **(B)** On-target editing efficiencies induced by each base editor (*i.e.*, editing at C_7_, equivalent to the installment of the m.3242G>A variant) **(C)** Average amplicon-wide off-target editing efficiencies. The horizontal dashed line corresponds to the mean of the untreated condition. **(D)** Average bystander editing efficiencies. **(E)** Normalized on-target-to-bystander editing ratios. TALE-free sDddA11: N- and C-termini of TALE-free, mitochondrially targeted, split DddA11–UGI. *TL1* α_L_DdCBE 3_CN_: base editor with split DddA11 in the C-to-N configuration, that is, left TALE–DddA11-C–UGI + right TALE–DddA11-N–UGI. *TL1* α_L_DdCBE 3_NC_: base editor with split DddA11 in the N-to-C configuration, that is, left TALE–DddA11-N–UGI + right TALE–DddA11-C–UGI. All other base editors are in the C-to-N orientation. All measurements were obtained via NGS and correspond to editing efficiencies in HEK293T cells 3 days post-transfection. All values and error bars represent the mean ± SD of *n* = 3 independent biological replicates.

In detail, we examined the performance of eight partially or fully modified base editors in installing the disease-associated variant m.3242G>A at the *TL1* locus in HEK293T cells. This base transition is equivalent to C-to-T editing at C_7_ in the *TL1* spacer region (Fig. 4E and Fig. 5A). For clarity, the partially modified pairs are denoted as α_L_DdCBEs, as only the left (L) arms contain unconstrained TALEs, and the fully modified pairs are referred to as αDdCBEs. Of note, *TL1* αDdCBE 3_NC_ corresponds to a pair with G1397-split DddA11 in the N-to-C configuration. All other pairs, including *TL1* αDdCBE 3_CN_, contain G1397-split DddA11 in the C-to-N orientation.

It is important to emphasize that, in this context, only C-to-T editing at C_7_ within the overall spacer region (*i.e.*, the installment of the m.3242G>A mutation) corresponds to on-target activity, while the conversion of other cytosines within a spacer is, by definition, bystander editing.^53,74^ Likewise, as in previous analyses, editing events outside of a spacer are considered off-target effects.^29,74^

In contrast to the low editing efficiencies observed at C_7_ with the DddA6-containing *TL1* base editor αA1-T2 (∼7%, Fig. 4F), its DddA11-containing counterpart, *TL1* α_L_DdCBE 2, edited C_7_ with substantially higher efficiencies (∼25%, Fig. 5A, B). However, while αA1-T2 resulted in amplicon-wide off-target editing at an average frequency of ∼0.02% (Fig. 4H, left), *TL1* α_L_DdCBE 2 installed off-target edits at an average frequency of ∼0.21% throughout the amplicon (Fig. 5C). Besides, both base editors led to distinct editing patterns (Fig. 4F, right, and Fig. 5A). Furthermore, TALE shifting enabled the screening of additional DddA11-containing pairs with varying performance metrics, as summarized in Figure 5.

Overall, *TL1*-specific, DddA11-containing α_L_DdCBEs and αDdCBEs led to on-target editing efficiencies ranging from ∼12% to ∼25% (Fig. 5B). In addition, *TL1* αDdCBEs can display improved levels of specificity compared with some *TL1* α_L_DdCBEs (Fig. 5C and Supplementary Fig. S5). In detail, *TL1* αDdCBEs 1, 2, and 3_CN_ resulted in significantly less average amplicon-wide off-target editing frequencies compared with *TL1* α_L_DdCBEs 1, 2, and 3, but greater or similar levels of off-target effects relative to *TL1* α_L_DdCBE 4. Similarly, *TL1* αDdCBE 3_NC_ was more specific than *TL1* α_L_DdCBEs 1 and 2, but not more specific than TL1 α_L_DdCBEs 3 and 4.

Regarding mutation patterns, C_7_ was efficiently edited by all pairs. In particular, *TL1* α_L_DdCBEs 1 and 2, which displayed promiscuous editing profiles, were the only pairs to edit C_3_ and C_4_. In contrast, more than half the activity of *TL1* α_L_DdCBE 3 and αDdCBE 3_CN_ corresponded to editing at C_7_ (Fig. 5A). Furthermore, despite differing in spacer length by just 1 bp relative to *TL1* αDdCBEs 1 and 2, *TL1* α_L_DdCBE 4 displayed increased activity at C_11_ and C_12_ (*p* < 0.0001 for all comparisons) and decreased editing at C_9_ (*p* < 0.001 for both comparisons). Moreover, likely due to their opposite orientations of split DddA11,^53^ *TL1* αDdCBEs 3_CN_ and 3_NC_ resulted in distinct mutation patterns.

In addition to assessing on- and off-target editing activities (Fig. 5B, C), we measured each base editor’s average bystander editing (Fig. 5D). Notably, *TL1* αDdCBE 3_CN_ displayed the lowest mean bystander editing efficiencies (*p* < 0.001 for all comparisons), while *TL1* α_L_DdCBE 2 showed the highest (*p* < 0.0001 for all comparisons). Subsequently, to quantify the performance of each base editor in efficiently and precisely installing the m.3242G>A variant, we calculated normalized on-target-to-bystander editing ratios (Fig. 5E), which are further informed by the off-target activities of each pair (Fig. 5C). Notably, *TL1* αDdCBE 3_CN_ outperformed all other pairs (*p* < 0.0001 for all comparisons), followed by TL1 α_L_DdCBE 3 (*p* < 0.001 for all comparisons).

In addition, we calculated normalized on-target-to-off-target editing ratios (Supplementary Fig. S6A). However, as bystander editing events are disregarded using this specific metric, these analyses do not fully reflect the performance of each base editor in precisely installing the m.3242G>A variant. Alternatively, we calculated normalized on-target-to-unintended (*i.e.*, bystander and off-target) editing ratios (Supplementary Fig. S6B). However, when compared with the normalized on-target-to-bystander editing ratios (Fig. 5E), the ratios in Supplementary Figure S6B overstate base editor performance. Thus, for applications in which a specific point mutation is desired, on-target-to-bystander editing ratios along with amplicon-wide specificity data can facilitate base editor selection.

In the context of DddA-derived base editors, these results collectively demonstrate that TALE shifting, that is, utilizing different sets of TALEs to access a single target site, can result in enhanced editing outcomes, both in terms of activity and cytosine selectivity within the spacer regions.

## DISCUSSION

In this study, we formally developed αDdCBEs for unconstrained mitochondrial base editing, which displayed improved performance compared with canonical DdCBEs across different target sites in human mtDNA *in vitro*. In addition, we demonstrated that αDdCBEs are compatible with DddA_tox_ and its engineered variants, DddA6 and DddA11. Furthermore, we validated TALE shifting with αDdCBEs as an approach to fine-tune base editing outcomes. This method enabled the definition of diverse spacers at a single target site, regardless of the most 5′ nucleotides available to the TALEs. In terms of practical applications, TALE shifting can be leveraged to modulate bystander editing.

Importantly, the relevance of the 5′-T constraint in the context of mitochondrially targeted, TALE-guided deaminases remained uncharacterized.^13,16,30–36^ By functionally exploring this design rule, we found that it often acts as a moderate limiting factor for efficient mtDNA editing with DdCBEs, rather than as an obligate requirement. In fact, several 5′-T-noncompliant DdCBEs efficiently edited their target sites, although occasionally at moderately decreased levels of on-target activity or increased frequencies of proximal off-target editing compared with αDdCBEs, which often outperformed their canonical counterparts (Supplementary Table S5).

In contrast to our findings, 5′-T compliance for optimal TALE activity in the nuclei of plant and animal cells is broadly supported by the frequent presence of thymine preceding the targets of natural TALE proteins, and by the standard guidelines for nuclear-targeted TALE-based technologies.^23–27,37–47^ These platforms include TALE scaffolds specifically engineered to circumvent the 5′-T constraint through amino acid substitutions in the TALE NTD.^52,75–80^ However, TALE sensitivity to the 5′-T constraint is influenced not only by the interaction between the canonical TALE NTD and a 5′-T on the target DNA,^39,52,76,79^ but also by the number and composition of repeats in the TALE protein.^44,77,81,82^

In particular, in an *in vitro* reporter assay, TALEs containing between 9.5 and 13.5 repeats showed a strong dependence on the 5′-T rule, while TALEs with 17.5 repeats were, in comparison, less influenced by this constraint.^77^ It should be noted that FusX-based technologies, including the DdCBEs in this study, are currently limited to TALEs with 14.5, 15.5, or 16.5 repeats,^49–51^ lengths associated with optimal TALE binding affinity in cell-free assays.^82^ Thus, given the number of repeats in the TALEs within the DdCBEs tested here, we speculate that the observed flexibility in the 5′-T rule might be partially explained by the TALE-length-dependent stringency of this requirement.^77^

Of note, the number of repeats in reported TALE-based technologies for mtDNA editing ranges from 8.5 to 19.5 repeats.^13,32–36,48,50,53,59,65,83,84^ Despite this variability in TALE length across systems, the effects of repeat number on the performance of TALE-based mitochondrial base editors remain unclear. Notably, our findings suggest that αDdCBEs containing TALEs with 14.5 or 15.5 repeats, particularly in 5′-T-noncompliant formats, can be moderately more active and specific than their canonical counterparts, suggesting an enhanced design flexibility under these conditions. In contrast, some αDdCBEs containing TALEs with 16.5 repeats resulted in less on-target activity than canonical DdCBEs.

Given these observations, it might be tempting to attempt to reduce the stringency of the 5′-T constraint in canonical DdCBEs by utilizing long TALEs (≥16.5 repeats). However, this potential strategy is yet to be thoroughly evaluated. Furthermore, each additional repeat in a single TALE protein introduces 34 amino acids (102 bp) to its sequence.^37,38^ For gene therapy applications, these increments in transgene size could hinder packaging into some delivery vectors.

Overall, we recommend the following approach for the use of αDdCBEs. First, target sites where αDdCBEs can be more applicable than DdCBEs can be identified by verifying whether 5′-T-compliant DdCBEs can be designed, based on standard design guidelines.^13,53^ Accordingly, we suggest using αDdCBEs if the target contains only one or no binding sites for 5′-T-compliant TALEs, as ignoring the 5′-T constraint can result in loss of performance. In detail, for αDdCBE design, we recommend spacers between 11 and 18 bp long, and TALEs with 14.5 or 15.5 repeats, irrespective of the most 5′ nucleotides of their targets. In addition, users may test αDdCBEs with short TALEs (8.5 to 13.5 repeats).

Of note, we suggest careful consideration to spacer composition, which can be modulated via TALE shifting with αDdCBEs. In general, narrow spacers can be defined to limit the number of bystander cytosines around a specific target nucleotide. Furthermore, αDdCBEs might facilitate testing of additional spacers at sites where only a limited set of 5′-T-compliant DdCBEs can be designed. Regarding deaminase domain selection, our work is consistent with the previous recommendations by Mok et al.^53^ Finally, we hypothesize that other effector domains, such as DddA homologs or chimeric deaminases,^16,32–36^ will also work well with unconstrained TALEs.

### Study limitations

In this study, αDdCBEs were developed using immortalized human cells *in vitro*. Further characterizations in clinically relevant cell types and *in vivo* models are needed to continue to validate our observations. Moreover, comparisons between DdCBEs and αDdCBEs were conducted at a limited number of sites, and although most measurements were obtained via NGS, some base editing activities were measured using Sanger sequencing, from which low-frequency variants cannot be detected. Likewise, although amplicon-wide analyses are highly informative,^29^ genome-wide surveys will enhance our understanding of the overall specificity of αDdCBEs.

## CONCLUSIONS

We determined that the TALE 5′-T constraint for mitochondrial base editing with DdCBEs can act as a moderate limiting factor, rather than a definitive requirement. For cases in which this design rule hampers canonical DdCBE performance, αDdCBEs can be more effective, particularly with 5′-T-noncompliant TALEs containing 14.5 or 15.5 repeats. In general, αDdCBEs provide an alternative for testing and can display increased on-target activity or reduced off-target editing relative to canonical DdCBEs.

## Data Availability

DdCBE and αDdCBE plasmids used in this study will be made available through Addgene. The FusX system for TALE assembly is available on Addgene (Kit # 1000000063). The general architecture of the base editors used in this study is detailed in the Supplementary Sequences. Main source data are provided as Supplementary Material. Raw chromatogram files (.ab1) from Sanger sequencing have been deposited in a repository publicly available at https://github.com/srcastillo/DdCBEs Raw paired-end read FASTQ files from high-throughput sequencing have been deposited in the NCBI Sequence Read Archive under accession code PRJNA1111950.

## References

[1] Anderson S, Bankier AT, Barrell BG, et al. Sequence and organization of the mitochondrial genome. Nature 1981;290(5806):457–465; doi: 10.1038/290457a0 7219534

[2] Taanman J-W. The mitochondrial genome: Structure, transcription, translation and replication. Biochim Biophys Acta 1999;1410(2):103–123; doi: 10.1016/S0005-2728(98)00161-3 10076021

[3] Stewart JB, Chinnery PF. The dynamics of mitochondrial DNA heteroplasmy: Implications for human health and disease. Nat Rev Genet 2015;16(9):530–542; doi: 10.1038/nrg3966 26281784

[4] Wallace DC, Chalkia D. Mitochondrial DNA genetics and the heteroplasmy conundrum in evolution and disease. Cold Spring Harb Perspect Biol 2013;5(11):a021220; doi: 10.1101/cshperspect.a021220 24186072 PMC3809581

[5] Spinelli JB, Haigis MC. The multifaceted contributions of mitochondria to cellular metabolism. Nat Cell Biol 2018;20(7):745–754; doi: 10.1038/s41556-018-0124-1 29950572 PMC6541229

[6] D’Souza AR, Minczuk M. Mitochondrial transcription and translation: Overview. Essays Biochem 2018;62(3):309–320; doi: 10.1042/EBC20170102 30030363 PMC6056719

[7] Nadler F, Lavdovskaia E, Richter-Dennerlein R. Maintaining mitochondrial ribosome function: The role of ribosome rescue and recycling factors. RNA Biol 2022;19(1):117–131; doi: 10.1080/15476286.2021.2015561 34923906 PMC8786322

[8] Gorman GS, Schaefer AM, Ng Y, et al. Prevalence of nuclear and mitochondrial DNA mutations related to adult mitochondrial disease. Ann Neurol 2015;77(5):753–759; doi: 10.1002/ana.24362 25652200 PMC4737121

[9] Naviaux RK. Mitochondrial DNA disorders. Eur J Pediatr 2000;159(S3):S219–S226; doi: 10.1007/PL00014407 11216904

[10] Taylor RW, Turnbull DM. Mitochondrial DNA mutations in human disease. Nat Rev Genet 2005;6(5):389–402; doi: 10.1038/nrg1606 15861210 PMC1762815

[11] Wallace DC. Mitochondrial DNA mutations in disease and aging. Environ Mol Mutagen 2010;51(5):440–450; doi: 10.1002/em.20586 20544884

[12] Pfeffer G, Majamaa K, Turnbull DM, et al. Treatment for mitochondrial disorders. Cochrane Database Syst Rev 2012;2012(4):CD004426; doi: 10.1002/14651858.CD004426.pub3 22513923 PMC7201312

[13] Mok BY, De Moraes MH, Zeng J, et al. A bacterial cytidine deaminase toxin enables CRISPR-free mitochondrial base editing. Nature 2020;583(7817):631–637; doi: 10.1038/s41586-020-2477-4 32641830 PMC7381381

[14] Falkenberg M, Hirano M. Editing the mitochondrial genome. N Engl J Med 2020;383(15):1489–1491; doi: 10.1056/NEJMcibr2025332 33027575 PMC8281826

[15] Kar B, Castillo SR, Sabharwal A, et al. Mitochondrial base editing: Recent advances towards therapeutic opportunities. Int J Mol Sci 2023;24(6):5798; doi: 10.3390/ijms24065798 36982871 PMC10056815

[16] Kim J-S, Chen J. Base editing of organellar DNA with programmable deaminases. Nat Rev Mol Cell Biol 2024;25(1):34–45; doi: 10.1038/s41580-023-00663-2 37794167

[17] Silva-Pinheiro P, Minczuk M. The potential of mitochondrial genome engineering. Nat Rev Genet 2022;23(4):199–214; doi: 10.1038/s41576-021-00432-x 34857922

[18] Sieber F, Duchêne A-M, Maréchal-Drouard L. Mitochondrial RNA import: From diversity of natural mechanisms to potential applications. Int Rev Cell Mol Biol 2011;287:145–190; doi: 10.1016/B978-0-12-386043-9.00004-9 21414588

[19] Gammage PA, Moraes CT, Minczuk M. Mitochondrial genome engineering: The revolution may not be CRISPR-ized. Trends Genet 2018;34(2):101–110; doi: 10.1016/j.tig.2017.11.001 29179920 PMC5783712

[20] Schmiderer L, Yudovich D, Oburoglu L, et al. Site-specific CRISPR-based mitochondrial DNA manipulation is limited by gRNA import. Sci Rep 2022;12(1):18687; doi: 10.1038/s41598-022-21794-0 36333335 PMC9636205

[21] Wright DA, Thibodeau-Beganny S, Sander JD, et al. Standardized reagents and protocols for engineering zinc finger nucleases by modular assembly. Nat Protoc 2006;1(3):1637–1652; doi: 10.1038/nprot.2006.259 17406455

[22] Maeder ML, Thibodeau-Beganny S, Sander JD, et al. Oligomerized Pool Engineering (OPEN): An “open-source” protocol for making customized zinc-finger arrays. Nat Protoc 2009;4(10):1471–1501; doi: 10.1038/nprot.2009.98 19798082 PMC2858690

[23] Mussolino C, Morbitzer R, Lütge F, et al. A novel TALE nuclease scaffold enables high genome editing activity in combination with low toxicity. Nucleic Acids Res 2011;39(21):9283–9293; doi: 10.1093/nar/gkr597 21813459 PMC3241638

[24] Reyon D, Tsai SQ, Khayter C, et al. FLASH assembly of TALENs for high-throughput genome editing. Nat Biotechnol 2012;30(5):460–465; doi: 10.1038/nbt.2170 22484455 PMC3558947

[25] DeFrancesco L. Erratum: Move over ZFNs. Nat Biotechnol 2012;30(1):112–112; doi: 10.1038/nbt0112-112c

[26] Baker M. Gene-editing nucleases. Nat Methods 2012;9(1):23–26; doi: 10.1038/nmeth.1807 22312637

[27] Kim Y, Kweon J, Kim A, et al. A library of TAL effector nucleases spanning the human genome. Nat Biotechnol 2013;31(3):251–258; doi: 10.1038/nbt.2517 23417094

[28] Lim K, Cho S-I, Kim J-S. Nuclear and mitochondrial DNA editing in human cells with zinc finger deaminases. Nat Commun 2022;13(1):366; doi: 10.1038/s41467-022-27962-0 35042880 PMC8766470

[29] Willis JCW, Silva-Pinheiro P, Widdup L, et al. Compact zinc finger base editors that edit mitochondrial or nuclear DNA *in vitro* and *in vivo*. Nat Commun 2022;13(1):7204. 36418298 10.1038/s41467-022-34784-7 PMC9684478

[30] Kotrys AV, Durham TJ, Guo XA, et al. Single-cell analysis reveals context-dependent, cell-level selection of mtDNA. Nature 2024;629(8011):458–466; doi: 10.1038/s41586-024-07332-0 38658765 PMC11078733

[31] Mahmood M, Liu EM, Shergold AL, et al. Mitochondrial DNA mutations drive aerobic glycolysis to enhance checkpoint blockade response in melanoma. Nat Cancer 2024;5(4):659–672; doi: 10.1038/s43018-023-00721-w 38286828 PMC11056318

[32] Sun H, Wang Z, Shen L, et al. Developing mitochondrial base editors with diverse context compatibility and high fidelity via saturated spacer library. Nat Commun 2023;14(1):6625; doi: 10.1038/s41467-023-42359-3 37857619 PMC10587121

[33] Cheng K, Li C, Jin J, et al. Engineering RsDddA as mitochondrial base editor with wide target compatibility and enhanced activity. Mol Ther Nucleic Acids 2023;34:102028; doi: 10.1016/j.omtn.2023.09.005 37744175 PMC10514076

[34] Wei Y, Jin M, Huang S, et al. Enhanced C-to-T and A-to-G base editing in mitochondrial DNA with engineered DdCBE and TALED. Adv Sci (Weinh) 2023;11(3):e2304113; doi: 10.1002/advs.202304113 37984866 PMC10797475

[35] Cho S-I, Lim K, Hong S, et al. Engineering TALE-linked deaminases to facilitate precision adenine base editing in mitochondrial DNA. Cell 2024;187(1):95–109.e26; doi: 10.1016/j.cell.2023.11.035 38181745

[36] Hu J, Sun Y, Li B, et al. Strand-preferred base editing of organellar and nuclear genomes using CyDENT. Nat Biotechnol 2023;42(6):936–945; doi: 10.1038/s41587-023-01910-9 37640945

[37] Moscou MJ, Bogdanove AJ. A simple cipher governs DNA recognition by TAL effectors. Science 2009;326(5959):1501–1501; doi: 10.1126/science.1178817 19933106

[38] Boch J, Scholze H, Schornack S, et al. Breaking the code of DNA binding specificity of TAL-type III effectors. Science 2009;326(5959):1509–1512. 19933107 10.1126/science.1178811

[39] Mak AN-S, Bradley P, Cernadas RA, et al. The crystal structure of TAL effector PthXo1 bound to its DNA target. Science 2012;335(6069):716–719; doi: 10.1126/science.1216211 22223736 PMC3427646

[40] Bogdanove AJ, Schornack S, Lahaye T. TAL effectors: Finding plant genes for disease and defense. Curr Opin Plant Biol 2010;13(4):394–401; doi: 10.1016/j.pbi.2010.04.010 20570209

[41] Cermak T, Doyle EL, Christian M, et al. Efficient design and assembly of custom TALEN and other TAL effector-based constructs for DNA targeting. Nucleic Acids Res 2011;39(12):e82; doi: 10.1093/nar/gkr218 21493687 PMC3130291

[42] Geiger-Schuller K, Mitra J, Ha T, et al. Functional instability allows access to DNA in longer Transcription Activator-Like effector (TALE) arrays. eElife 2019;8:e38298; doi: 10.7554/eLife.38298 PMC6461438 30810525

[43] Cuculis L, Abil Z, Zhao H, et al. TALE proteins search DNA using a rotationally decoupled mechanism. Nat Chem Biol 2016;12(10):831–837; doi: 10.1038/nchembio.2152 27526029

[44] Rogers JM, Barrera LA, Reyon D, et al. Context influences on TALE–DNA binding revealed by quantitative profiling. Nat Commun 2015;6(1):7440; doi: 10.1038/ncomms8440 26067805 PMC4467457

[45] Bogdanove AJ, Voytas DF. TAL effectors: Customizable proteins for DNA targeting. Science 2011;333(6051):1843–1846; doi: 10.1126/science.1204094 21960622

[46] Becker S, Boch J. TALE and TALEN genome editing technologies. Gene Genome Ed 2021;2:100007; doi: 10.1016/j.ggedit.2021.100007

[47] Mahfouz MM, Li L, Shamimuzzaman M, et al. De novo-engineered Transcription Activator-Like Effector (TALE) hybrid nuclease with novel DNA binding specificity creates double-strand breaks. Proc Natl Acad Sci USA 2011;108(6):2623–2628; doi: 10.1073/pnas.1019533108 21262818 PMC3038751

[48] Mok YG, Lee JM, Chung E, et al. Base editing in human cells with monomeric DddA-TALE fusion deaminases. Nat Commun 2022;13(1):4038. 35821233 10.1038/s41467-022-31745-y PMC9276701

[49] Ma AC, McNulty MS, Poshusta TL, et al. FusX: A rapid one-step transcription activator-like effector assembly system for genome science. Hum Gene Ther 2016;27(6):451–463; doi: 10.1089/hum.2015.172 26854857 PMC4931509

[50] Sabharwal A, Kar B, Restrepo-Castillo S, et al. The FusX TALE base editor (FusXTBE) for rapid mitochondrial DNA programming of human cells *in vitro* and zebrafish disease models in vivo. CRISPR J 2021;4(6):799–821; doi: 10.1089/crispr.2021.0061 34847747 PMC8742272

[51] Kar B, Sabharwal A, Restrepo-Castillo S, et al. An optimized FusX assembly-based technique to introduce mitochondrial TC-to-TT variations in human cell lines. STAR Protoc 2022;3(2):101288; doi: 10.1016/j.xpro.2022.101288 35496789 PMC9038556

[52] Lamb BM, Mercer AC, Barbas CF. Directed evolution of the TALE N-terminal domain for recognition of all 5′ bases. Nucleic Acids Res 2013;41(21):9779–9785; doi: 10.1093/nar/gkt754 23980031 PMC3834825

[53] Mok BY, Kotrys AV, Raguram A, et al. CRISPR-free base editors with enhanced activity and expanded targeting scope in mitochondrial and nuclear DNA. Nat Biotechnol 2022;40(9):1378–1387; doi: 10.1038/s41587-022-01256-8 35379961 PMC9463067

[54] Ye J, Coulouris G, Zaretskaya I, et al. Primer-BLAST: A tool to design target-specific primers for polymerase chain reaction. BMC Bioinformatics 2012;13(1):134; doi: 10.1186/1471-2105-13-134 22708584 PMC3412702

[55] Tourmen Y, Baris O, Dessen P, et al. Structure and chromosomal distribution of human mitochondrial pseudogenes. Genomics 2002;80(1):71–77; doi: 10.1006/geno.2002.6798 12079285

[56] Skerra A. Phosphorothioate primers improve the amplification of DNA sequences by DNA polymerases with proofreading activity. Nucleic Acids Res 1992;20(14):3551–3554; doi: 10.1093/nar/20.14.3551 1641322 PMC334000

[57] Clement K, Rees H, Canver MC, et al. CRISPResso2 provides accurate and rapid genome editing sequence analysis. Nat Biotechnol 2019;37(3):224–226; doi: 10.1038/s41587-019-0032-3 30809026 PMC6533916

[58] Merkel D. Docker: Lightweight Linux containers for consistent development and deployment. Linux J 2014;2014(239):2.

[59] Lee S, Lee H, Baek G, et al. Precision mitochondrial DNA editing with high-fidelity DddA-derived base editors. Nat Biotechnol 2023;41(3):378–386; doi: 10.1038/s41587-022-01486-w 36229610 PMC10017512

[60] Lei Z, Meng H, Liu L, et al. Mitochondrial base editor induces substantial nuclear off-target mutations. Nature 2022;606(7915):804–811; doi: 10.1038/s41586-022-04836-5 35551512

[61] Kluesner MG, Nedveck DA, Lahr WS, et al. EditR: A method to quantify base editing from Sanger sequencing. CRISPR J 2018;1(3):239–250; doi: 10.1089/crispr.2018.0014 31021262 PMC6694769

[62] Goto Y, Nonaka I, Horai S. A mutation in the tRNALeu(UUR) gene associated with the MELAS subgroup of mitochondrial encephalomyopathies. Nature 1990;348(6302):651–653; doi: 10.1038/348651a0 2102678

[63] Manwaring N, Jones MM, Wang JJ, et al. Population prevalence of the MELAS A3243G mutation. Mitochondrion 2007;7(3):230–233; doi: 10.1016/j.mito.2006.12.004 17300999

[64] Li D, Liang C, Zhang T, et al. Pathogenic mitochondrial DNA 3243A>G mutation: From genetics to phenotype. Front Genet 2022;13:951185; doi: 10.3389/fgene.2022.951185 36276941 PMC9582660

[65] Yi Z, Zhang X, Tang W, et al. Strand-selective base editing of human mitochondrial DNA using mitoBEs. Nat Biotechnol 2024;42(3):498–509; doi: 10.1038/s41587-023-01791-y 37217751 PMC10940147

[66] Gattermann N, Wulfert M, Junge B, et al. Ineffective hematopoiesis linked with a mitochondrial tRNA mutation (G3242A) in a patient with myelodysplastic syndrome. Blood 2004;103(4):1499–1502; doi: 10.1182/blood-2003-07-2446 14576046

[67] Mimaki M, Hatakeyama H, Ichiyama T, et al. Different effects of novel mtDNA G3242A and G3244A base changes adjacent to a common A3243G mutation in patients with mitochondrial disorders. Mitochondrion 2009;9(2):115–122; doi: 10.1016/j.mito.2009.01.005 19460299

[68] Wortmann SB, Champion MP, Van Den Heuvel L, et al. Mitochondrial DNA m.3242G>A mutation, an under diagnosed cause of hypertrophic cardiomyopathy and renal tubular dysfunction? Eur J Med Genet 2012;55(10):552–556; doi: 10.1016/j.ejmg.2012.06.002 22781753

[69] Wong L-JC, Chen T, Wang J, et al. Interpretation of mitochondrial tRNA variants. Genet Med 2020;22(5):917–926; doi: 10.1038/s41436-019-0746-0 31965079

[70] Ardissone A, Ferrera G, Lamperti C, et al. Phenotyping mitochondrial DNA-related disease in childhood: a cohort study of 150 patients. Eur J Neurol 2023;30(7):2079–2091; doi: 10.1111/ene.15814 37038312

[71] Kirino Y, Goto Y, Campos Y, et al. Specific correlation between the wobble modification deficiency in mutant tRNAs and the clinical features of a human mitochondrial disease. Proc Natl Acad Sci U S A 2005;102(20):7127–7132; doi: 10.1073/pnas.0500563102 15870203 PMC1129107

[72] Yakubovskaya E, Mejia E, Byrnes J, et al. Helix unwinding and base flipping enable human MTERF1 to terminate mitochondrial transcription. Cell 2010;141(6):982–993; doi: 10.1016/j.cell.2010.05.018 20550934 PMC2887341

[73] Karasik A, Wilhelm CA, Fierke CA, et al. Disease-associated mutations in mitochondrial precursor tRNAs affect binding, m1R9 methylation, and tRNA processing by mtRNase P. RNA 2021;27(4):420–432; doi: 10.1261/rna.077198.120 33380464 PMC7962481

[74] Rees HA, Liu DR. Base editing: Precision chemistry on the genome and transcriptome of living cells. Nat Rev Genet 2018;19(12):770–788; doi: 10.1038/s41576-018-0059-1 30323312 PMC6535181

[75] Tsuji S, Futaki S, Imanishi M. Creating a TALE protein with unbiased 5′-T binding. Biochem Biophys Res Commun 2013;441(1):262–265; doi: 10.1016/j.bbrc.2013.10.060 24148249

[76] Doyle EL, Hummel AW, Demorest ZL, et al. TAL effector specificity for base 0 of the DNA target is altered in a complex, effector- and assay-dependent manner by substitutions for the tryptophan in cryptic repeat –1. Jeltsch A. ed. PLoS One 2013;8(12):e82120; doi: 10.1371/journal.pone.0082120 24312634 PMC3849474

[77] Schreiber T, Bonas U. Repeat 1 of TAL effectors affects target specificity for the base at position zero. Nucleic Acids Res 2014;42(11):7160–7169; doi: 10.1093/nar/gku341 24792160 PMC4066769

[78] Hubbard BP, Badran AH, Zuris JA, et al. Continuous directed evolution of DNA-binding proteins to improve TALEN specificity. Nat Methods 2015;12(10):939–942; doi: 10.1038/nmeth.3515 26258293 PMC4589463

[79] Richter A, Streubel J, Boch J. TAL effector DNA-binding principles and specificity. In: TALENs. Methods in Molecular Biology. (Kühn R, Wurst W, Wefers B. eds.) Humana Press: New York, NY; 2016. 10.1007/978-1-4939-2932-0_2 26443210

[80] Sun N, Liang J, Abil Z, et al. Optimized TAL effector nucleases (TALENs) for use in treatment of sickle cell disease. Mol Biosyst 2012;8(4):1255–1263; doi: 10.1039/c2mb05461b 22301904

[81] Meckler JF, Bhakta MS, Kim M-S, et al. Quantitative analysis of TALE–DNA interactions suggests polarity effects. Nucleic Acids Res 2013;41(7):4118–4128; doi: 10.1093/nar/gkt085 23408851 PMC3627578

[82] Rinaldi FC, Doyle LA, Stoddard BL, et al. The effect of increasing numbers of repeats on TAL effector DNA binding specificity. Nucleic Acids Res 2017;45(11):6960–6970; doi: 10.1093/nar/gkx342 28460076 PMC5499867

[83] Cho S-I, Lee S, Mok YG, et al. Targeted A-to-G base editing in human mitochondrial DNA with programmable deaminases. Cell 2022;185(10):1764–1776.e12; doi: 10.1016/j.cell.2022.03.039 35472302

[84] Silva-Pinheiro P, Mutti CD, Van Haute L, et al. A library of base editors for the precise ablation of all protein-coding genes in the mouse mitochondrial genome. Nat Biomed Eng 2022;7(5):692–703; doi: 10.1038/s41551-022-00968-1 36470976 PMC10195678

