# Supplementary material for: Unconstrained Precision Mitochondrial Genome Editing with αDdCBEs
Source: Hum Gene Ther. 2024 Oct 14;35(19-20):798–813. doi: 10.1089/hum.2024.073 (PMC11511777; doi:10.1089/hum.2024.073)
Supplement: Supplementary Figure S1 [file hum.2024.073_supplementary_figure_s1.pdf]

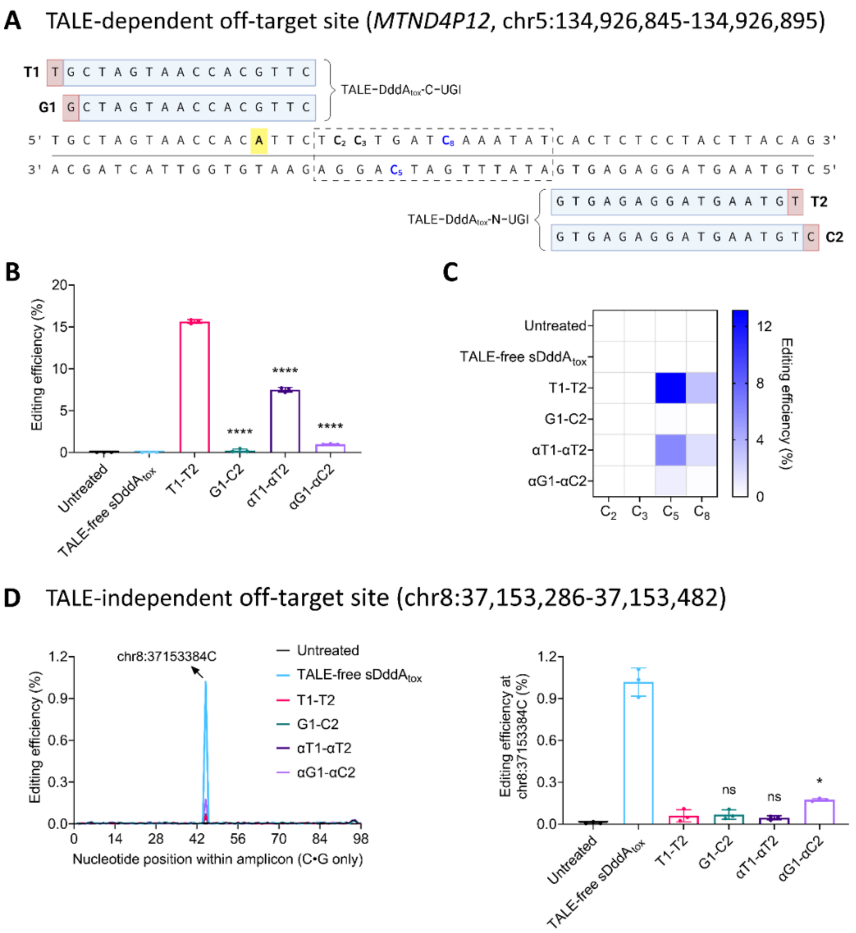

Supplementary Figure S1. Nuclear off-targets effects induced by *ND4* DdCBEs and αDdCBEs. (A) TALE-dependent off-target site at the nuclear mitochondrial pseudogene *MTND4P12*, which differs from the mitochondrial sequence targeted by the indicated *ND4* base editors by a single nucleotide mismatch (highlighted in yellow). The TALE off-target sequences are shown in the blue rectangles, and the nucleotides immediately upstream of these sequences are indicated in the red boxes. Cytosines in the pseudo-spacer (dashed box) are numbered from the 3' end of the left TALE off-target sequence. Edited cytosines are highlighted in blue. **(B)** Overall off-target editing efficiencies and **(C)** corresponding mutation patterns at *MTND4P12*. **(D)** Left, amplicon-wide visualization of the editing efficiencies at a TALE-independent off-target site, which shares no sequence similarity with the *ND4* DdCBE on-target sequence. Right, off-target editing at chr8:37153384C (hg38). TALE-free sDddA$_{tox}$: N- and C-termini of TALE-free, mitochondrially targeted, split DddA$_{tox}$–UGI. T1-T2: 5'-T-compliant *ND4* DdCBE pair; G1-C2: 5'-T-noncompliant *ND4* DdCBE pair; αT1-αT2: 5'-T-compliant *ND4* αDdCBE pair; αG1-αC2: 5'-T-noncompliant *ND4* αDCBE pair. All measurements were obtained via NGS and correspond to editing efficiencies in HEK293T cells 3 days post-transfection. Values and error bars in **(B)** through **(D)** represent the mean ± s.d. of n = 3 independent biological replicates. Displayed statistical significances in **(B)** and **(D)** were determined by comparing against the T1-T2 condition. *P<0.05; ****P<0.0001; ns (not significant), P>0.05 by two-tailed unpaired t test in GraphPad Prism 10.
