# Supplementary material for: Unconstrained Precision Mitochondrial Genome Editing with αDdCBEs
Source: Hum Gene Ther. 2024 Oct 14;35(19-20):798–813. doi: 10.1089/hum.2024.073 (PMC11511777; doi:10.1089/hum.2024.073)
Supplement: Supplementary Figure S2 [file hum.2024.073_supplementary_figure_s2.pdf]

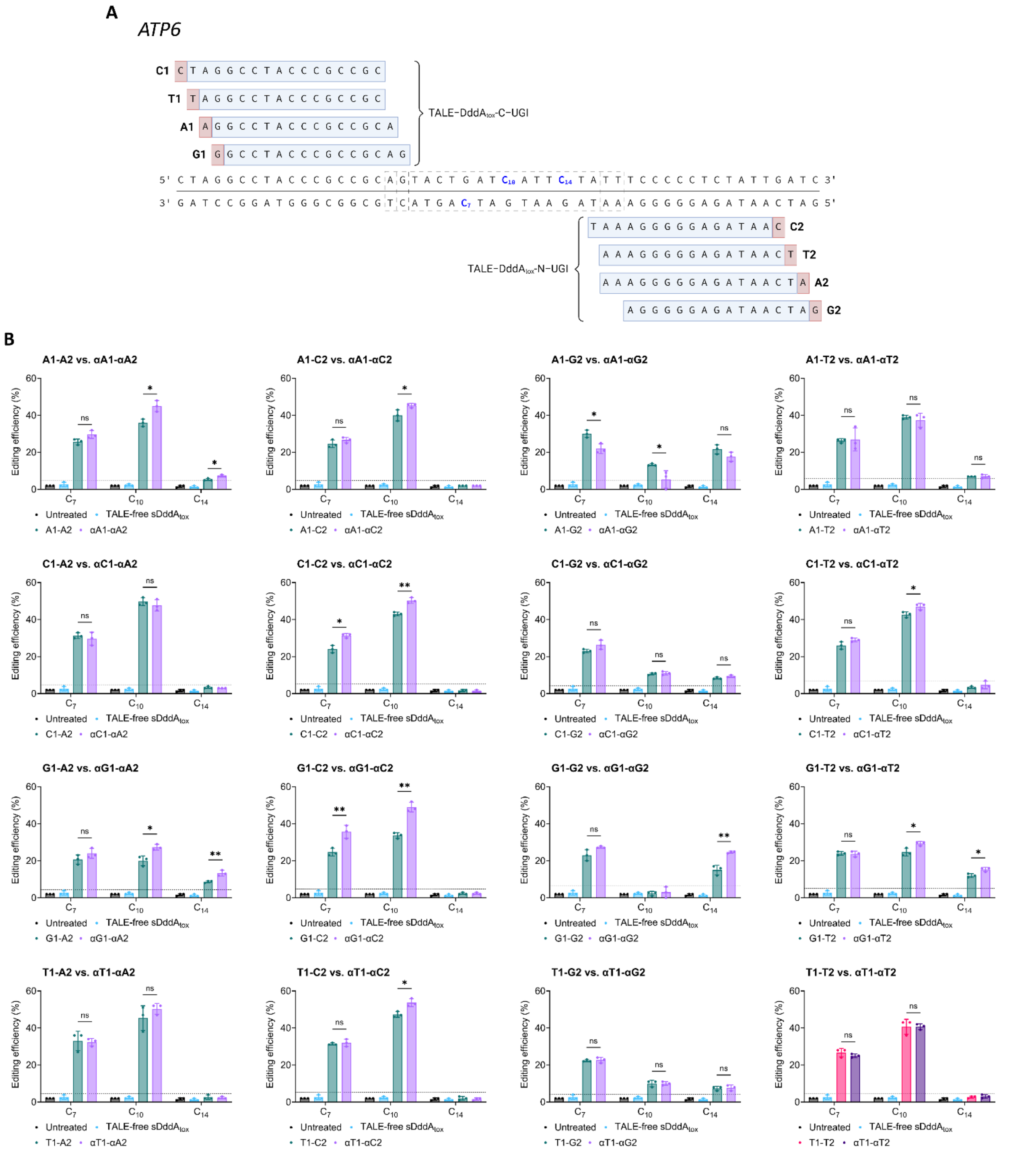

Supplementary Figure S2. All N1-N2 vs. αN1-αN2 comparisons at *ATP6*. (A) Schematic of the mitochondrial on-target site within *ATP6*. The sequences targeted by the TALE repeat arrays are shown in the blue rectangles, and the nucleotides immediately upstream of these sequences are indicated in the red boxes. Each base editor arm is denominated as N1/N2 (where 'N' represents A, C, G, or T) depending on the corresponding most 5' nucleotide of its TALE binding site and whether it constitutes the left ('1') or the right ('2') arm of the construct. αN1-αN2: αDdCBE pairs. The spacer regions are indicated by the dashed boxes. Cytosines that were consistently edited across conditions are numbered and highlighted

in blue from the 3' end of the T1 arm. **(B)** Comparisons between the editing efficiencies induced by DdCBEs and αDdCBEs in every possible N1-N2 combination, from A1-A2 (top left) to T1-T2 (bottom right, shown in red and purple to maintain the color scheme used in other figures). TALE-free sDddA$_{tox}$: N- and C-termini of TALE-free, mitochondrially targeted, split DddA$_{tox}$–UGI constructs. All measurements were obtained via Sanger sequencing trace decomposition with EditR and correspond to editing efficiencies in HEK293T cells 3 days post-transfection. Values and error bars represent the mean ± s.d. of n = 3 independent biological replicates. The horizontal dashed lines correspond to critical percent values, obtained from EditR with a P-value cutoff of 0.01, above which base editing estimates are significantly different from background. *P<0.05; **P<0.01; ns (not significant), P>0.05 by two-tailed unpaired t test in GraphPad Prism 10.
