# Supplementary material for: Unconstrained Precision Mitochondrial Genome Editing with αDdCBEs
Source: Hum Gene Ther. 2024 Oct 14;35(19-20):798–813. doi: 10.1089/hum.2024.073 (PMC11511777; doi:10.1089/hum.2024.073)
Supplement: Supplementary Figure S3 [file hum.2024.073_supplementary_figure_s3.pdf]

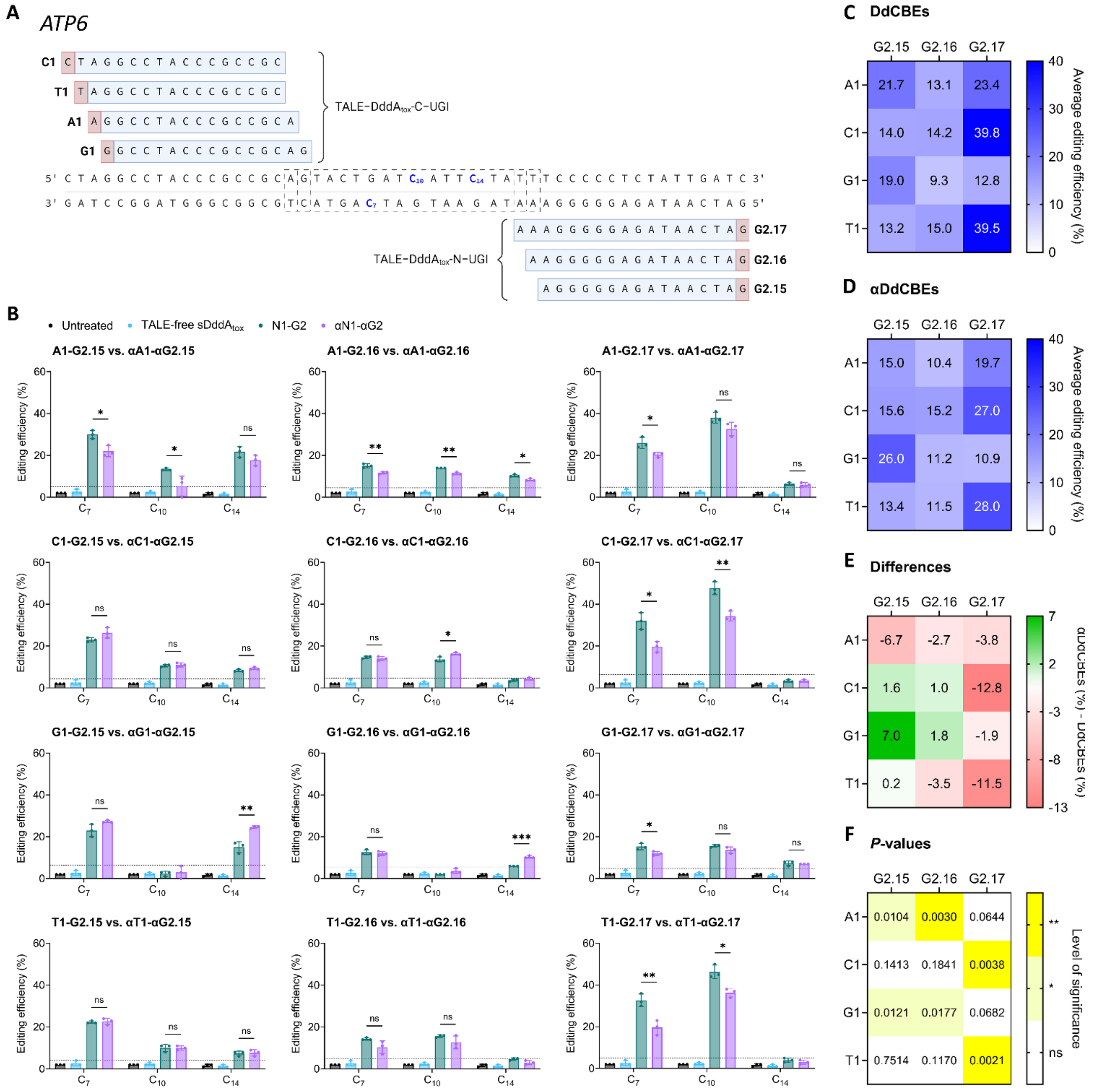

Supplementary Figure S3. Additional N1-G2 vs. αN1-αG2 comparisons at *ATP6*. (A) Schematic of the mitochondrial on-target site within *ATP6*. The sequences targeted by the TALE repeat arrays are shown in the blue rectangles, and the nucleotides immediately upstream of these sequences are indicated in the red boxes. Each base editor arm is denominated as N1/G2 (where 'N' represents A, C, G, or T) depending on the corresponding most 5' nucleotide of its TALE binding site and whether it constitutes the left ('1') or the right ('2') arm of the construct. αN1-αG2: αDdCBE pairs. The spacer regions are indicated by the dashed boxes. Cytosines that were consistently edited across conditions are numbered and highlighted in blue from the 3' end of the T1 arm. G2.15/αG2.15, which target a 15 bp long sequence preceded by a guanine, corresponds to the right DdCBE/αDdCBE arm used in the main comparisons in **Fig. 3** and **Supplementary Fig. S2**. Similarly, G2.16/αG2.16 and G2.17/αG2.17 correspond to right DdCBE/αDdCBE arms that target 16 and 17 bp long sequences, respectively, both preceded by the same guanine. **(B)**

Comparisons between the editing efficiencies induced by DdCBEs and αDdCBEs in every possible N1-G2 combination. All measurements were obtained via Sanger sequencing trace decomposition with EditR and correspond to editing efficiencies in HEK293T cells 3 days post-transfection. Values and error bars represent the mean ± s.d. of n = 3 independent biological replicates. The horizontal dashed lines correspond to critical percent values, obtained from EditR with a P-value cutoff of 0.01, above which base editing estimates are significantly different from background. TALE-free sDddA$_{tox}$: N- and C-termini of TALE-free, mitochondrially targeted, split DddA$_{tox}$–UGI. **(C)**, **(D)** Average DdCBE- and αDdCBE-induced editing efficiencies. **(E)** Differences between the average editing efficiencies induced by αDdCBEs and DdCBEs. **(F)** Corresponding P-values for statistical comparisons between the average editing efficiencies displayed in **(C)** and **(D)**. *P<0.05; **P<0.01; ns (not significant), P>0.05 by two-tailed unpaired t test in GraphPad Prism 10.
