# Supplementary material for: Unconstrained Precision Mitochondrial Genome Editing with αDdCBEs
Source: Hum Gene Ther. 2024 Oct 14;35(19-20):798–813. doi: 10.1089/hum.2024.073 (PMC11511777; doi:10.1089/hum.2024.073)
Supplement: Supplementary Figure S4 [file hum.2024.073_supplementary_figure_s4.pdf]

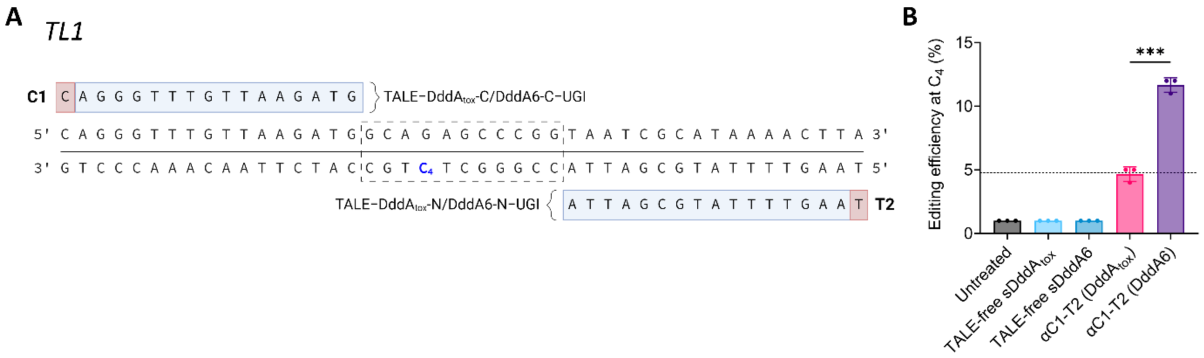

Supplementary Figure S4. $DddA_{tox}$- vs. DddA6-containing *TL1* α_LDdCBEs. (A) Schematic of the mitochondrial on-target site within *TL1* and preliminary α_LDdCBEs (only the left arm contains an unconstrained) tested to select an optimal effector domain. The sequences targeted by the TALE repeat arrays are shown in the blue rectangles, and the nucleotides immediately upstream of these sequences are indicated in the red boxes. The spacer is indicated by the dashed box. C_4, highlighted in blue, which is numbered relative to its position from the 3' end of the left TALE target sequence, corresponds to the only efficiently edited cytosine within the spacer. **(B)** Editing efficiencies at C_4 across conditions. TALE-free $sDddA_{tox}$/sDddA6: N- and C-termini of TALE-free, mitochondrially targeted, split $DddA_{tox}$/DddA6–UGI. αC1-T2 ($DddA_{tox}$): $DddA_{tox}$-containing *TL1* α_LDdCBE pair. αC1-T2 (DddA6): DddA6-containing *TL1* α_LDdCBE pair. All measurements were obtained via Sanger sequencing trace decomposition with EditR and correspond to editing efficiencies in HEK293T cells 3 days post-transfection. Values and error bars represent the mean ± s.d. of $n = 3$ independent biological replicates. The horizontal dashed line corresponds to a critical percent value, obtained from EditR with a *P*-value cutoff of 0.01, above which base editing estimates are significantly different from background. ***$P<0.001$ by two-tailed unpaired *t* test in GraphPad Prism 10.
