# Supplementary material for: Unconstrained Precision Mitochondrial Genome Editing with αDdCBEs
Source: Hum Gene Ther. 2024 Oct 14;35(19-20):798–813. doi: 10.1089/hum.2024.073 (PMC11511777; doi:10.1089/hum.2024.073)
Supplement: Supplementary Figure S5 [file hum.2024.073_supplementary_figure_s5.pdf]

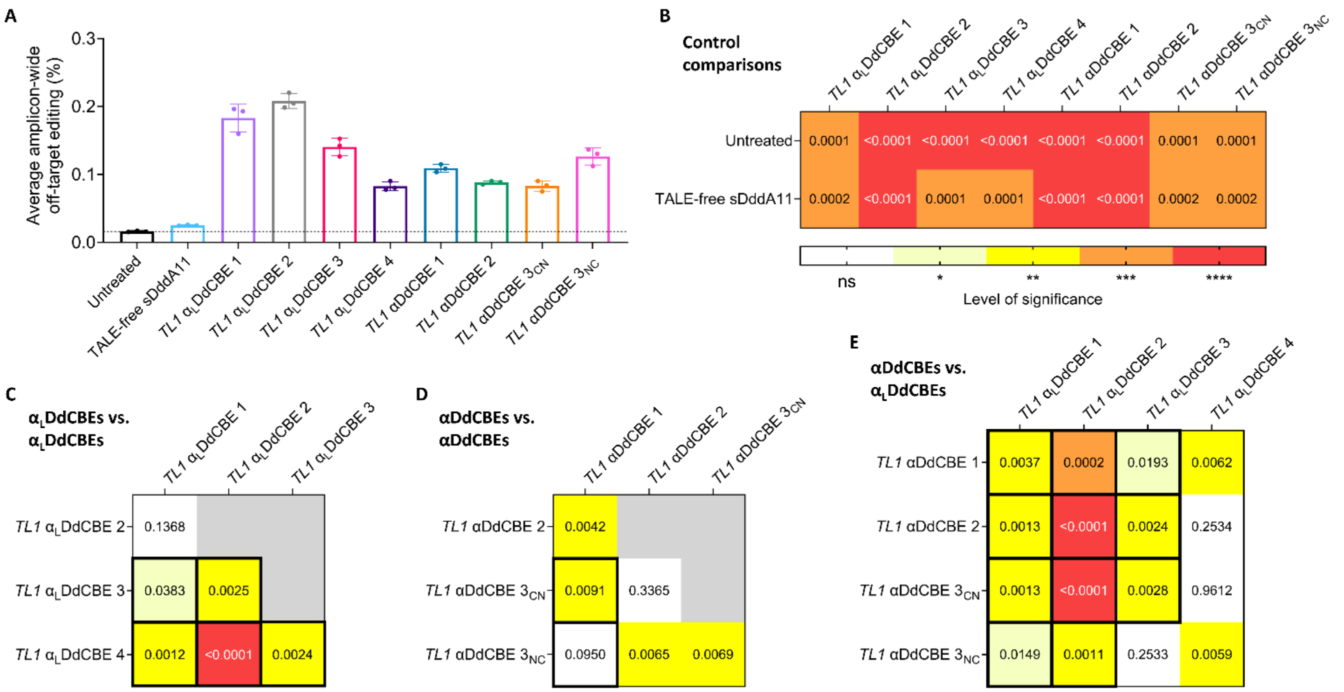

Supplementary Figure S5. Comparisons between the average proximal off-target effects of *TL1*-specifc, DddA11-containing α_LDdCBEs and αDdCBEs. (A) Average amplicon-wide off-target editing frequencies. The horizontal dashed line indicates the mean of the untreated condition. All measurements were obtained via NGS and correspond to editing efficiencies 3 days post-transfection. All values and error bars represent the mean ± s.d. of n = 3 independent biological replicates. For clarity, the corresponding P-values for all comparisons are divided as follows: **(B)** against the negative controls (untreated and TALE-free sDddA11), **(C)** between α_LDdCBEs, **(D)** between αDdCBEs, and **(E)** between α_LDdCBEs and αDdCBEs. The color bar in **(B)** represents the scale used for all the heatmaps. The gray cells in the symmetric heatmaps in **(C)** and **(D)** indicate duplicated values, which are omitted to avoid redundancy and simplify the visualization of the data. The black squares in panels **(C)** through **(E)** highlight comparisons in which the base editor on the row is significantly more specific (i.e., results in less average amplicon-wide off-target editing) than the base editor on the column. TALE-free sDddA11: N- and C-termini of TALE-free, mitochondrially targeted, split DddA11–UGI. *TL1* α_LDdCBE 3_{CN}: base editor with split DddA11 in the C-to-N configuration, i.e., left TALE–DddA11-C–UGI + right TALE–DddA11-N–UGI. *TL1* α_LDdCBE 3_{NC}: base editor with split DddA11 in the N-to-C configuration, i.e., left TALE–DddA11-N–UGI + right TALE–DddA11-C–UGI. All other base editors are in the C-to-N orientation. *$P<0.05$; **$P<0.01$; ***$P<0.001$; ****$P<0.0001$; ns (not significant), $P>0.05$ by two-tailed unpaired t test in GraphPad Prism 10.
