# Supplementary material for: Unconstrained Precision Mitochondrial Genome Editing with αDdCBEs
Source: Hum Gene Ther. 2024 Oct 14;35(19-20):798–813. doi: 10.1089/hum.2024.073 (PMC11511777; doi:10.1089/hum.2024.073)
Supplement: Supplementary Figure S6 [file hum.2024.073_supplementary_figure_s6.pdf]

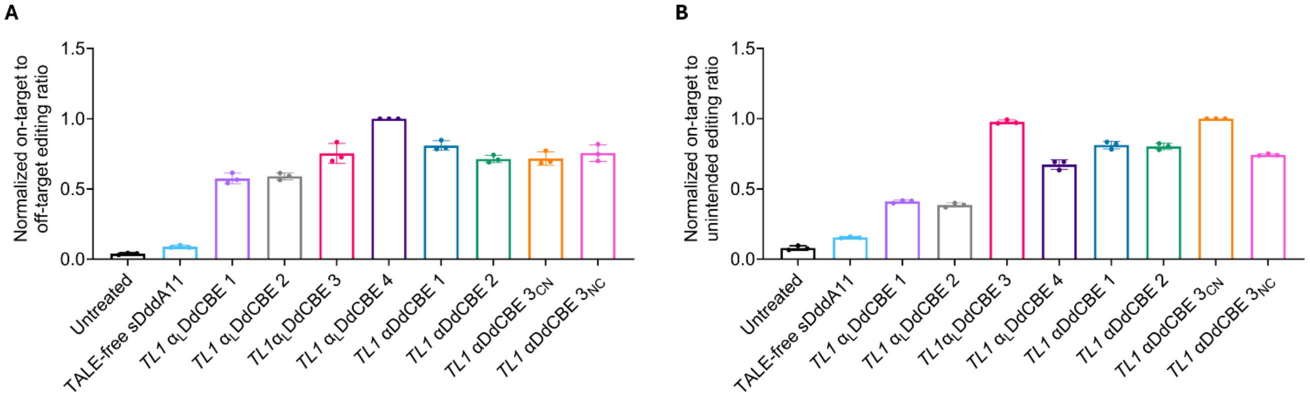

Supplementary Figure S6. Additional metrics for *TL1*-specifc, DddA11-containing α_LDdCBEs and αDdCBEs. (A) Normalized on-target to off-target editing ratios, where on-target corresponds to C-to-T editing at C_7 within the *TL1* spacer region, and off-target corresponds to the average amplicon-wide off-target editing frequencies. **(B)** Normalized on-target to unintended editing ratios, where unintended refers to both off-target and bystander editing frequencies. TALE-free sDddA11: N- and C-termini of TALE-free, mitochondrially targeted, split DddA11–UGI. *TL1* α_LDdCBE 3_{CN}: base editor with split DddA11 in the C-to-N configuration, i.e., left TALE–DddA11-C–UGI + right TALE–DddA11-N–UGI. *TL1* α_LDdCBE 3_{NC}: base editor with split DddA11 in the N-to-C configuration, i.e., left TALE–DddA11-N–UGI + right TALE–DddA11-C–UGI. All other base editors are in the C-to-N orientation. All measurements were obtained via NGS and correspond to editing efficiencies 3 days post-transfection. All values and error bars represent the mean ± s.d. of *n* = 3 independent biological replicates
