# Supplementary material for: Unconstrained Precision Mitochondrial Genome Editing with αDdCBEs
Source: Hum Gene Ther. 2024 Oct 14;35(19-20):798–813. doi: 10.1089/hum.2024.073 (PMC11511777; doi:10.1089/hum.2024.073)
Supplement: Supplementary Table S1 [file hum.2024.073_supplementary_table_s1.pdf]

Supplementary Table S1. Summary of subcloning strategies to generate all backbone plasmids. Vector and insert plasmids used to clone all FusX-compatible DdCBE backbones used in this study. Insert plasmid pUC57 FusX, containing a 'FusX cassette', was obtained through a gene synthesis service (GenScript). The FusX cassette consists of a fragment of the canonical TALE N-terminal domain (TALE NT-T), a BsmBI restriction site, the components necessary for blue-white screening (i.e., CAP binding site–lac promoter–lac operator–lacZα), an additional BsmBI restriction site, and a fragment of the TALE C-terminal domain. TALE NT-αN: unconstrained TALE N-terminal domain.[1–7]

Backbone plasmid	Vector plasmid			Insert plasmid			Enzymes
	Source	Origin	Replaced	Source	Origin	Inserted	
DdCBE 1397C	ND5.1-DdCBE-right side TALE	Dr. David Liu (Broad Institute)[1]	ND5.1 right TALE	pUC57 FusX	This study	FusX cassette	PstI and BamHI
DdCBE 1397N	ND4-DdCBE-right side TALE	Addgene plasmid no. 157843[1]	ND4 right TALE	pUC57 FusX	This study	FusX cassette	PstI and BamHI
αDdCBE 1397C	ND6-DdCBE-right side TALE	Addgene plasmid no. 157841[1]	ND6 right TALE	pUC57 FusX	This study	FusX cassette	PstI and BamHI
αDdCBE 1397N	DdCBE 1397N	This study	TALE NT-T	αDdCBE 1397C	This study	TALE NT-αN	NheI and PstI
DdCBE DddA6/11 1397C	DdCBE 1397N	This study	DddAtox 1397C	ND5.2-Left DdCBE-G1397C-T1413I	Dr. David Liu (Broad Institute)[6]	DddAtox 1397C (T1413I)	BamHI and Bsu36I
DdCBE DddA6 1397N	ND5.2-Right TALE-G1397-N-DddA6	Dr. David Liu (Broad Institute)[6]	ND5.2 right TALE	pUC57 FusX	This study	FusX cassette	PstI and BamHI
DdCBE DddA11 1397N	ND5.2-Right TALE-G1397-N-DddA11	Dr. David Liu (Broad Institute)[6]	ND5.2 right TALE	pUC57 FusX	This study	FusX cassette	PstI and BamHI
αDdCBE DddA6/11 1397C	DdCBE DddA6/11 1397C	This study	TALE NT-T	αDdCBE 1397C	This study	TALE NT-αN	NheI and PstI
αDdCBE DddA6 1397N	DdCBE DddA6 1397N	This study	TALE NT-T	ND6-DdCBE-right side TALE	Addgene plasmid no. 157841[6]	TALE NT-αN	SacI and PstI
αDdCBE DddA11 1397N	αDdCBE 1397C	This study	$DddA_{tox}$ 1397C	ND5.2-Right TALE-G1397-N-DddA11	Dr. David Liu (Broad Institute)[6]	DddA11 1397N	BamHI and Bsu36I
mDdCBE	DdCBE 1397N	This study	$DddA_{tox}$ 1397N	pCMV-ND1 Right-GSVG-UGI	Addgene plasmid no. 187413[7]	$DddA_{tox}$ GSVG	BamHI and Bsu36I
