# Supplementary material for: Unconstrained Precision Mitochondrial Genome Editing with αDdCBEs
Source: Hum Gene Ther. 2024 Oct 14;35(19-20):798–813. doi: 10.1089/hum.2024.073 (PMC11511777; doi:10.1089/hum.2024.073)
Supplement: Supplementary Table S2 [file hum.2024.073_supplementary_table_s2.pdf]

Supplementary Table S2. TALE binding sites of all base editors used in this study. For simplicity, arms with the same TALE target sequence are grouped together. In the *TL1* section, α/α_L-1/2/3/4 (left/right) refers to the base editors in **Fig. 5**. E.g., 'α_L-1 (left)' corresponds to the left arm of *TL1* α_LDdCBE 1. For each TALE, its corresponding 5'-N nucleotide is specified (N_0), as well as the target sequence of its central repeat domain (CRD). Also, the plasmids utilized for FusX-based assembly are listed on the right.

Target	Base editor arm	TALE target seq. (5'-to-3')		FusX plasmids for assembly[2–4]						
		N_0	CRD target seq.	X1	X2	X3	X4	B2	B3	LR
ATP6	A1/αA1	A	GGCCTACCCGCCGCA	42	29	22	38	10	-	NI
	A2/αA2	A	TCAATAGAGGGGGAAA	53	13	35	43	-	33	NI
	C1/αC1	C	TAGGCCTACCCGCCGC	51	38	50	23	-	23	HD
	C2/αC2	C	AATAGAGGGGGAAAT	4	9	43	41	1	-	NG
	G1/αG1	G	GCCTACCCGCCGCAG	38	50	23	23	5	-	NN
	G2.15/αG2.15 (G2/αG2)	G	ATCAATAGAGGGGGA	14	4	9	43	11	-	NI
	G2.16/αG2.16	G	ATCAATAGAGGGGGAA	14	4	9	43	-	41	NI
	G2.17/αG2.17	G	ATCAATAGAGGGGGAAA	14	4	9	43	-	41	11
	T1/αT1	T	AGGCCTACCCGCCGC	11	24	6	26	7	-	HD
	T2/αT2	T	CAATAGAGGGGGAAA	17	51	11	43	1	-	NI
CO1	A2/αA2	A	GGTGTTGGTATAGAA	44	48	44	13	9	-	NI
	C1/αC1	C	TTCTTCGACCCCGCCG	62	62	34	22	-	38	NN
	T1/αT1	T	TCTTCGACCCCGCCG	56	55	6	23	6	-	NN
	T2/αT2	T	AGGTGTTGGTATAGAA	11	60	59	52	-	9	NI
ND2	A2/αA2	A	GCTGGGTTTGGTTTA	40	43	64	44	16	-	NI
	C1/αC1	C	TTATCCATCATAGCAGG	61	54	14	13	-	37	16
	T1/αT1	T	ATCCATCATAGCAGG	14	20	20	10	3	-	NN
	T2/αT2	T	AGCTGGGTTTGGTTTA	10	59	48	59	-	64	NI
ND4	C2/αC2	C	TGTAAGTAGGAGAGTG	60	3	51	35	-	12	NN
	G1/αG1	G	CTAGTAACCACGTTC	29	45	6	7	16	-	HD
	T1/αT1	T	GCTAGTAACCACGTTC	40	12	2	18	-	48	HD
	T2/αT2	T	GTAAGTAGGAGAGTG	45	12	11	9	12	-	NN
TC	mA1/A1/αA1	A	AGCCCCGGCAGGTTT	10	22	42	11	16	-	NG
	mT2/T2/αT2	T	ATTGAATTGCAAATT	16	33	63	17	4	-	NG
TL1	A1/αA1/α_L-2 (left)	A	ACAGGGTTTGTTAAG	5	43	64	48	1	-	NN
	αC1/α_L-3/α_L-4/α-1 (left)	C	AGGGTTTGTTAAGATG	11	48	60	49	-	36	NN
	T2/αT2/α_L-1/α_L-2/α_L-3 (right)	T	AAGTTTTATGCGATTA	3	64	52	39	-	16	NI
	α_L-1 (left)	G	AACAGGGTTTGTTAAG	2	11	48	60	-	49	NN
	α_L-4 (right)	T	TTAAGTTTTATGCGA	61	12	64	15	7	-	NI
	α-1/α-2 (right)	T	TTTAAGTTTTATGCG	64	3	64	52	10	-	NN
	α-2 (left)	A	GGGTTTGTTAAGATG	43	64	48	3	4	-	NN
	α-3_{CN}/α-3_{NC} (left)	C	AGGGTTTGTTAAGAT	11	48	60	49	9	-	NG
	α-3_{CN}/α-3_{NC} (right)	G	TTTTAAGTTTTATGC	64	49	48	61	15	-	HD
