# Supplementary material for: Unconstrained Precision Mitochondrial Genome Editing with αDdCBEs
Source: Hum Gene Ther. 2024 Oct 14;35(19-20):798–813. doi: 10.1089/hum.2024.073 (PMC11511777; doi:10.1089/hum.2024.073)
Supplement: Supplementary Table S3 [file hum.2024.073_supplementary_table_s3.pdf]

Supplementary Table S3. Primers for cloning TALE-free constructs and colony PCR. Plasmids encoding TALE-free, mitochondrially targeted split deaminase domain–UGI were generated via site-directed mutagenesis (SDM) with the primers indicated below, which were designed using NEBaseChanger (NEB) and synthesized as standard DNA oligos (IDT).

Template	Primers		TALE-free construct
	Name	Sequence (5'-to-3')	
DdCBE 1397N<br>DdCBE DddA6 1397N<br>DdCBE DddA11 1397N	DddAN F	GGATCCGGCAGCTACGCC	TALE-free $DddA_{tox}$-N–UGI<br>TALE-free DddA6-N–UGI<br>TALE-free DddA11-N–UGI
	3xFLAG R1	CATCTTGTCATCGTCATCCTTGTAATCGATG	
DdCBE 1397C<br>DdCBE DddA6/11 1397C	DddAC F	GGATCCGCCATTCCAGTG	TALE-free $DddA_{tox}$-C–UGI<br>TALE-free DddA6/11-C–UGI
	3xFLAG R2	CATCTTGTCATCGTCATCCTTG	

Additionally, colony PCR in FusX-based assembly[4] was conducted with the standard DNA oligos (IDT) indicated below. For assemblies with backbone plasmids DdCBE DddA6 1397N or DdCBE DddA11 1397N, FusX F2 was used. For assemblies with all other backbone plasmids, FusX F1 was used.

Primers	
Name	Sequence (5'-to-3')
FusX F1	CTACCCATGAAGCGATTGTG
FusX F2	CCACCCATGAAGCTATTGTG
FusX R	ATCCACCCAGGCCTTTCTTC
