# Supplementary material for: Unconstrained Precision Mitochondrial Genome Editing with αDdCBEs
Source: Hum Gene Ther. 2024 Oct 14;35(19-20):798–813. doi: 10.1089/hum.2024.073 (PMC11511777; doi:10.1089/hum.2024.073)
Supplement: Supplementary Table S4 [file hum.2024.073_supplementary_table_s4.pdf]

Supplementary Table S4. Primers for Sanger sequencing and NGS.

Target	Primers	
	Name	Sequence (5'-3')
ATP6	*ATP6* F	caccataattacccccatact*c
	ATP6 R	gtcctttagtgttgtgtatgg*t
	ATP6 BC1 F	ACACTCTTTCCCTACACGACGCTCTTCCGATCT**ATCACG**caccataattacccccatact*c
	ATP6 BC2 F	ACACTCTTTCCCTACACGACGCTCTTCCGATCT**CGATGT**caccataattacccccatact*c
	ATP6 BC3 F	ACACTCTTTCCCTACACGACGCTCTTCCGATCT**TTAGGC**caccataattacccccatact*c
	ATP6 NGS R	GACTGGAGTTCAGACGTGTGCTCTTCCGATCTgtcctttagtgttgtgtatgg*t
CO1	*CO1* NGS F	ACACTCTTTCCCTACACGACGCTCTTCCGATCTttctccttacacctagcaggt*g
	CO1 BC1 R	GACTGGAGTTCAGACGTGTGCTCTTCCGATCT**ATCACG**gctcgtgtgtctacgtctatt
	CO1 BC2 R	GACTGGAGTTCAGACGTGTGCTCTTCCGATCT**CGATGT**gctcgtgtgtctacgtctatt
	CO1 BC3 R	GACTGGAGTTCAGACGTGTGCTCTTCCGATCT**TTAGGC**gctcgtgtgtctacgtctatt
ND2	*ND2* BC1 F	ACACTCTTTCCCTACACGACGCTCTTCCGATCT**ATCACG**ggttacccaaggcaccc
	ND2 BC2 F	ACACTCTTTCCCTACACGACGCTCTTCCGATCT**CGATGT**ggttacccaaggcaccc
	ND2 BC3 F	ACACTCTTTCCCTACACGACGCTCTTCCGATCT**TTAGGC**ggttacccaaggcaccc
	ND2 NGS R	GACTGGAGTTCAGACGTGTGCTCTTCCGATCTggtgcgagatagtagtagggt*c
ND4	*ND4* BC1 F	ACACTCTTTCCCTACACGACGCTCTTCCGATCT**ATCACG**ttctcataatcgcccacgg*g
	ND4 BC2 F	ACACTCTTTCCCTACACGACGCTCTTCCGATCT**CGATGT**ttctcataatcgcccacgg*g
	ND4 BC3 F	ACACTCTTTCCCTACACGACGCTCTTCCGATCT**TTAGGC**ttctcataatcgcccacgg*g
	ND4 NGS R	GACTGGAGTTCAGACGTGTGCTCTTCCGATCTggttgagggataggaggag*a
TC	*TC* NGS F	ACACTCTTTCCCTACACGACGCTCTTCCGATCTcctacctatctccccttttat*a
	TC BC1 R	GACTGGAGTTCAGACGTGTGCTCTTCCGATCT**ATCACG**ccaatgtctttgtggtttgtag
	TC BC2 R	GACTGGAGTTCAGACGTGTGCTCTTCCGATCT**CGATGT**ccaatgtctttgtggtttgtag
	TC BC3 R	GACTGGAGTTCAGACGTGTGCTCTTCCGATCT**TTAGGC**ccaatgtctttgtggtttgtag
TL1	*TL1* F	cgtttgttcaacgattaaag
	TL1 R	agcgaagggttgtagtagcc
	TL1 BC1 F	ACACTCTTTCCCTACACGACGCTCTTCCGATCT**ATCACG**cgtttgttcaacgattaaag
	TL1 BC2 F	ACACTCTTTCCCTACACGACGCTCTTCCGATCT**CGATGT**cgtttgttcaacgattaaag
	TL1 BC3 F	ACACTCTTTCCCTACACGACGCTCTTCCGATCT**TTAGGC**cgtttgttcaacgattaaag
	TL1 NGS R	GACTGGAGTTCAGACGTGTGCTCTTCCGATCTagcgaagggttgtagtagcc
MTND4P12	TASd F1	ggataggaggaggataggggata
	TASd R1	cggcgcagtcattctcata*g
	TASd BC1 F2	ACACTCTTTCCCTACACGACGCTCTTCCGATCT**ATCACG**ttaatgtggtggctgagcg
	TASd BC2 F2	ACACTCTTTCCCTACACGACGCTCTTCCGATCT**CGATGT**ttaatgtggtggctgagcg
	TASd BC3 F2	ACACTCTTTCCCTACACGACGCTCTTCCGATCT**TTAGGC**ttaatgtggtggctgagcg
	TASd NGS R2	GACTGGAGTTCAGACGTGTGCTCTTCCGATCTcaagcctcactaatctcgcc
chr8:37153286-37153482 (hg38)	TASi BC1 F	ACACTCTTTCCCTACACGACGCTCTTCCGATCT**ATCACG**ggagaaacgtcacggtatgc
	TASi BC2 F	ACACTCTTTCCCTACACGACGCTCTTCCGATCT**CGATGT**ggagaaacgtcacggtatgc
	TASi BC3 F	ACACTCTTTCCCTACACGACGCTCTTCCGATCT**TTAGGC**ggagaaacgtcacggtatgc
	TASi NGS R	GACTGGAGTTCAGACGTGTGCTCTTCCGATCTggtggtcaacaattctcactgc

Non-bold, capital letters in the primer sequences correspond to partial Illumina® adapter sequences. Bold, capital letters correspond to barcodes for multiplexing. Lower case letters correspond the annealing sites of the primers. Asterisks (*) denote phosphorothioate bonds.[8]

ATP6 F and *ATP6* R were used to obtain the Sanger sequencing data in **Fig. 3**, **Supplementary Fig. S2**, and **Supplementary Fig. S3**. *TL1* F and *TL1* R were used to obtain the next-generation sequencing (NGS) data in **Fig. 5**, and the Sanger sequencing data in **Supplementary Fig. S4**. TASd F1 and TASd R1 were used for the first PCR in the nested PCR strategy to amplify the TALE-dependent off-target site within *MTND4P12* (**Supplementary Fig. S1**). All other primers were used to obtain the NGS data in the rest of the figures. All Sanger sequencing and NGS reactions were done through Genewiz.
