# Supplementary material for: Unconstrained Precision Mitochondrial Genome Editing with αDdCBEs
Source: Hum Gene Ther. 2024 Oct 14;35(19-20):798–813. doi: 10.1089/hum.2024.073 (PMC11511777; doi:10.1089/hum.2024.073)
Supplement: Supplementary Table S5 [file hum.2024.073_supplementary_table_s5.pdf]

Supplementary Table S5. Summary of on- and off-target editing efficiencies at sites including comparisons between DdCBEs and αDdCBEs. Each table corresponds to a gene used for comparisons between 5'-T-compliant and 5'-T-noncompliant DdCBEs and αDdCBEs. In this context, 'on-target' refers to the overall targeted editing activity within a spacer, while 'off-target' refers to the average amplicon-wide editing efficiency outside of a spacer. The standard DdCBE conditions for each gene are highlighted in yellow. The green and orange highlights indicate measurements from base editors that show statistically significant differences compared to the standard. Green indicates an improvement relative to the standard, while orange indicates either less on-target or more off-target activities compared to the standard. The corresponding levels of statistical significance for each comparison can be found in their respective figures (specified at the top of each table).

***ND2* (Figs. 1 and 2)**	**Mean ± s.d. (%, *n* = 3)**	
	**On-target**	**Off-target**
**Untreated**	0.13 ± 0.01	< 0.01
**TALE-free sDddA$_{\text{tox}}$**	0.27 ± 0.03	0.013 ± 0.001
**T1-T2†**	31.88 ± 1.20	0.045 ± 0.008
**C1-A2†**	21.08 ± 0.30	0.025 ± 0.003
**αT1-αT2‡**	28.95 ± 1.12	0.026 ± 0.002
**αC1-αA2‡**	30.08 ± 0.30	0.027 ± 0.001

***ND4* (Figs. 1 and 2)**	**Mean ± s.d. (%, *n* = 3)**	
	**On-target**	**Off-target**
**Untreated**	0.07 ± 0.02	< 0.01
**TALE-free sDddA$_{\text{tox}}$**	0.18 ± 0.01	0.013 ± 0.002
**T1-T2**	23.78 ± 0.39	0.019 ± 0.001
**G1-C2†**	16.80 ± 0.63	0.016 ± 0.001
**αT1-αT2‡**	22.50 ± 1.46	0.016 ± 0.001
**αG1-αC2‡**	22.97 ± 1.64	0.016 ± 0.002

***ATP6* (Figs. 1 and 2)**	**Mean ± s.d. (%, *n* = 3)**	
	**On-target**	**Off-target**
**Untreated**	0.09 ± 0.01	< 0.01
**TALE-free sDddA$_{\text{tox}}$**	0.18 ± 0.01	0.014 ± 5x10^{-4}
**T1-T2†**	35.75 ± 2.23	0.027 ± 0.003
**C1-A2†**	28.39 ± 0.29	0.022 ± 0.001
**αT1-αT2‡**	31.90 ± 0.39	0.017 ± 3×10^{-4}
**αC1-αA2‡**	30.10 ± 1.06	0.016 ± 0.001

***CO1* (Figs. 1 and 2)**	**Mean ± s.d. (%, *n* = 3)**	
	**On-target**	**Off-target**
**Untreated**	0.11 ± 0.01	0.011 ± 2x10^{-4}
**TALE-free sDddA$_{\text{tox}}$**	0.28 ± 0.02	0.020 ± 0.001
**T1-T2†**	19.87 ± 0.59	0.051 ± 0.001
**C1-A2**	18.88 ± 0.40	0.043 ± 0.002
**αT1-αT2**	22.21 ± 0.79	0.050 ± 0.002
**αC1-αA2‡**	19.81 ± 0.59	0.037 ± 0.001

***TC* (Fig. 4)**	**Mean ± s.d. (%, *n* = 3)**	
	**On-target**	**Off-target**
**Untreated**	0.09 ± 0.02	0.010 ± 0.001
**TALE-free sDddA$_{\text{tox}}$**	0.27 ± 0.02	0.020 ± 0.001
**mA1**	23.67 ± 0.34	0.272 ± 0.005
**mT2**	14.15 ± 0.09	0.166 ± 0.005
**A1-T2†**	54.38 ± 2.02	0.106 ± 0.008
**αA1-T2**	53.85 ± 0.61	0.082 ± 0.001

***TL1* (Fig. 4)**	**Mean ± s.d. (%, *n* = 3)**	
	**On-target**	**Off-target**
**Untreated**	0.10 ± 0.01	< 0.01
**TALE-free sDddA6**	0.10 ± 0.01	< 0.01
**A1-T2†**	2.34 ± 0.07	0.019 ± 0.002
**αA1-T2‡**	6.83 ± 0.22	0.020 ± 0.001
**A1-αT2†**	2.25 ± 0.18	0.020 ± 0.001
**αA1-αT2‡**	7.08 ± 0.23	0.023 ± 0.001

A1-αT2	56.23 ± 0.91	0.080 ± 0.002
αA1-αT2‡	48.67 ± 1.35	0.064 ± 0.002

The thick borders emphasize fully or partially modified base editors that resulted in either similar on-target and less off-target activity than the standard, or more on-target and similar off-target activity than the standard.

†Underperforming base editor/s (mA1 and mT2 in the *TC* table are excluded), ‡top-performing base editor/s, as determined by two-tailed unpaired *t* tests (GraphPad Prism 10) between the normalized on- to off-target editing ratios of the base editors at each target site. Note that two base editors may be classified as underperformers or top-performers, as there is no statistically significant difference between their performances.

TALE-free sDddAtox/sDddA6: N- and C-termini of TALE-free, mitochondrially targeted, split DddAtox/DddA6–UGI. T1-T2: 5'-T-compliant DdCBE pairs. V1-V2 (where 'V' represents a non-T nucleotide): 5'-T-noncompliant DdCBE pairs. αT1-αT2: 5'-T-compliant αDdCBE pairs. αV1-αV2: 5'-T-noncompliant αDdCBE pairs. mA1: 5'-T-noncompliant monomeric DdCBE (mDdCBE) control. mT2: 5'-T-compliant mDdCBE control. A1-T2: canonical DdCBE pair. αA1-T2 and A1-αT2: partially modified pairs. αA1-αT2: αDdCBE pair. All measurements were obtained via NGS and correspond to editing efficiencies in HEK293T cells 3 days post-transfection. The values represent the mean ± s.d. of n = 3 independent biological replicates.

Supplementary Sequences. Architecture of base editors used in this study

All DdCBE and αDdCBE arms, and monomeric DdCBEs (mDdCBEs) used in this study have the general architecture (from N- to C-terminus): COX8A MTS–3xFLAG–TALE NTD–TALE repeat array–TALE CTD–2aa linker–split deaminase domain or full-length $\mathrm{DddA}_{\mathrm{tox}}$ GSVG–4aa linker–UGI–ATP5B 3'UTR.

Below are the amino acid sequences of the two TALE N-terminal domains (TALE NT-T and TALE NT-αN) the general form of a FusX-based TALE repeat array, and the TALE C-terminal domain.[1,2,5] The differences between the TALE N-terminal domains are underlined in their respective sequences. The repeat variable diresidue (RVD) motifs are shown as XX in the TALE repeat array sequence. Similarly, the three dots (…) separate the first repeat from the last half repeat. All repeats in between have the same composition as the first repeat. The RVD-to-nucleotide correspondence is NI = A, HD = C, NN = G, and NG = T.[2]

TALE NT-T[1,5]

DIADLRTLGYSQQQQEKIKPKVRSTVAQHHEALVGHGFTHAHIVALSQHPAALGTVAVKYQDMIAALPEA
THEAIVGVGKQWSGARALEALLTVAGELRGPPLQLDTGQLLKIAKRGGVTAVEAVHAWRNALTGAPLN

TALE NT-αN[1,5]

DIADLRTLGYSQQQQEKIKPKVRSTVAQHHEALVGHGFTHAHIVALSQHPAALGTVAVKYQDMIAALPEA
THEAIVGVGKRGAGARALEALLTVAGELRGPPLQLDTGQLLKIAKRGGVTAVEAVHAWRNALTGAPLN

FusX-based TALE repeat array[2]

LTPDQVVAIASXXGGKQALETVQRLLPVLCQDHG…LTPDQVVAIASXXGGKQALE

TALE C-terminal domain[1]

SIVAQLSRPDPALAALTNDHLVALACLGGRPALDAVKKGLG

The amino acid sequences of the remaining components are available in their respective sources.[1,6,7]

Supplementary References

1. Mok BY, De Moraes MH, Zeng J, et al. A bacterial cytidine deaminase toxin enables CRISPR-free mitochondrial base editing. Nature 2020;583(7817):631–637; doi: 10.1038/s41586-020-2477-4.

2. Ma AC, McNulty MS, Poshusta TL, et al. FusX: a rapid one-step transcription activator-like effector assembly system for genome science. Hum Gene Ther 2016;27(6):451–463; doi: 10.1089/hum.2015.172.

3. Sabharwal A, Kar B, Restrepo-Castillo S, et al. The FusX TALE base editor (FusXTBE) for rapid mitochondrial DNA programming of human cells in vitro and zebrafish disease models in vivo. CRISPR J 2021;4(6):799–821; doi: 10.1089/crispr.2021.0061.

4. Kar B, Sabharwal A, Restrepo-Castillo S, et al. An optimized FusX assembly-based technique to introduce mitochondrial TC-to-TT variations in human cell lines. STAR Protoc 2022;3(2):101288; doi: 10.1016/j.xpro.2022.101288.

5. Lamb BM, Mercer AC, Barbas CF. Directed evolution of the TALE N-terminal domain for recognition of all 5’ bases. Nucleic Acids Res 2013;41(21):9779–9785; doi: 10.1093/nar/gkt754.

6. Mok BY, Kotrys AV, Raguram A, et al. CRISPR-free base editors with enhanced activity and expanded targeting scope in mitochondrial and nuclear DNA. Nat Biotechnol 2022;40(9):1378–1387; doi: 10.1038/s41587-022-01256-8.

7. Mok YG, Lee JM, Chung E, et al. Base editing in human cells with monomeric DddA-TALE fusion deaminases. Nat Commun 2022;13(1):4038.

8. Skerra A. Phosphorothioate primers improve the amplification of DNA sequences by DNA polymerases with proofreading activity. Nucleic Acids Res 1992;20(14):3551–3554; doi: doi.org/10.1093/nar/20.14.3551.
